# The deubiquitinating enzyme USP4 regulates BRCA1 stability and function

**DOI:** 10.1038/s41523-024-00641-7

**Published:** 2024-05-11

**Authors:** Xueyuan Guo, Yanfang Ma, Ting Zhang, Runyu Liu, Fen Chang, Xingyue Yan, Tianyun Yu, Pengfei Wu, Qin Li, Luzheng Xu, Junyi Duan, Li Li, Yanrong Su, Genze Shao

**Affiliations:** 1 Department of Cell Biology, School of Basic Medical Sciences, Peking University Health Science Center, Beijing, 100191 China; 2 Center of Medical and Health Analysis, Peking University Health Science Center, Beijing, 100191 China; 3 The Irma H. Russo, MD Breast Cancer Research Laboratory, Fox Chase Cancer Center-Temple University Health System, Philadelphia, PA 19111 USA

**Keywords:** Breast cancer, Homologous recombination, Ubiquitylation

## Abstract

BRCA1 plays a suppressive role in breast tumorigenesis. Ubiquitin-dependent degradation is a common mechanism that regulates BRCA1 protein stability, and several ubiquitin ligases involved have been identified. However, the deubiquitinating enzyme for BRCA1 remains less defined. Here, we report that the deubiquitinase USP4 interacts with, deubiquitinates and stabilizes BRCA1, maintaining the protein level of BRCA1. USP4 knockdown results in a decreased BRCA1 protein level, impairment in homologous recombination mediated double-stranded break repair, and increased genome instability, and confers resistance to DNA damage-inducing agents and PARP inhibitors. Ectopic expression of USP4 stabilizes BRCA1 and reverse the effects caused by USP4 knockdown. Moreover, USP4 is low expressed in human breast cancer tissues and its low expression correlates with poorer survival of patients. Furthermore, we identified several loss-of-function mutations of USP4 in human gynecological cancers, the catalytic activity of which or their interaction with BRCA1 is disrupted. Together, we reveal that USP4 is a deubiquitinase for BRCA1. USP4 positively regulates the stability and function of BRCA1 through de-ubiquitination, and plays important role in the suppression of breast cancer.

## Introduction

*BRCA1* is one of the most frequently mutated genes in human breast and ovarian cancers. Genetic susceptibility is responsible for about 5–10% of all breast cancer cases, in which germ-line mutations of BRCA1 account for 50% of them^1,2^. Besides, growing evidence shows that BRCA1 protein level reduction also plays a role in sporadic breast and ovarian cancers, which account for the vast majority of all cases^3–5^. BRCA1 serves as a tumor suppressor to participate in many important biological processes including homologous recombination (HR) -mediated DNA damage repair, S and G2/M cell cycle checkpoint, chromatin remodeling, replication fork protection and transcriptional regulation^6–12^, and loss of these functions either by mutation or by reduction in protein level is believed to be responsible for the accumulation of genomic instability and even tumorigenesis.

BRCA1 protein stability and activity are tightly regulated at multiple levels. First, BRCA1 is stabilized when it forms complex with BARD1. It is estimated that approximately 70% of total cellular BRCA1 exists as BRCA1/BARD1 heterodimers and displays enhanced E3 ligase activity^13^. Second, cellular BRCA1 protein expression is regulated at transcription level, and other mechanisms such as microRNA-based regulation and alternative splicing also play a role in influencing BRCA1 mRNA level and protein expression^14–18^. Third, BRCA1 protein level is also regulated by post-translational modifications, for example, ubiquitylation. Several E3 ubiquitin ligases including HERC2, FBXO44, HUWE1 and DCAF8L1 have been identified to directly target BRCA1 for polyubiquitylation and degradation through ubiquitin proteasome system (UPS)^19–22^. Besides, some BRCA1 interacting proteins such as TUSC4 or CTSS could also regulate BRCA1 level, probably through blocking the binding between HERC2 and BRCA1 (TUSC4)^23^, or cleaving BRCT of BRCA1 and thus facilitating its degradation by UPS (CTSS)^24^.

Ubiquitylation is a reversible and balanced process. E3 ligase mediated degradation of many proteins can be reversed by deubiquitinating enzymes (DUBs). Nearly 98 DUBs in human genome are categorized by their catalytic domain architecture, including metalloprotease JAB1/MPN/Mov34 (JAMM), cysteine proteases ubiquitin C-terminal hydroxylase (UCH), Machado-Joseph disease protease (MJD), Ovarian Tumor protease (OTU), Motif interacting with Ub-containing novel DUB family (MINDY), and ubiquitin-specific protease (USP) families, in which USPs account for the largest proportion containing nearly 60 members^25–27^. Emerging evidence demonstrates that USPs participate in the regulation of HR-directed DNA repair, in which BRCA1 plays a critical role^28,29^. Many DUBs including USP13, USP15, USP26, USP37, USP48, BRCC36, USP9X and BAP1 have been demonstrated to regulate HR repair, probably through a mechanism that impacts BRCA1 related function^30–33^, however, none of them have been demonstrated to be the specific DUB for the de-ubiquitination of BRCA1. For example, BAP1 was initially identified as a BRCA1 interacting protein^34^, but later study showed that it only binds BARD1 and perturbs the formation of BRCA1/BARD1. Moreover, the DUB activity of BAP1 is not required to inhibit the E3 ligase activity of BRCA1^35^. USP15, one of the USP4 paralogue, was shown to regulate HR through targeting BARD1 at the BRCT domain, and promote BARD1-HP1γ interaction, resulting in BRCA1/BARD1 retention at DSBs. BRCC36, USP13, USP26, USP37 and USP48 all participate in regulating BRCA1-mediated HR, but they seemly have little effect on the stability of BRCA1^32,36–39^. USP9X do physically interact with BRCA1 and depletion can lead to significant decreased BRCA1 protein level^30^, however, because it was reported to regulate BRCA1 mRNA abundance through a mechanism independent of its DUB catalytic activity^40^, it is not yet clear whether it is a bona fide DUB of BRCA1.

USP4 is a member of USP family and is involved in the regulation of multiple signaling pathways by deubiquitinating various substrates. For instance, USP4 is reported to participate in the control of p53-related signaling through deubiquitinating ARF-BP1^41^, the suppression of canonical Wnt signaling pathway through deubiquitinating TCF4^42^, the activation of TGF-β signaling through deubiquitinating substrates like TβR-I and SMAD4^43,44^ and the inhibition of NF-κB pathway through deubiquitinating mediators like TRAF2, TRAF6 and TAK1^45,46^. In addition, USP4 also plays an important role in the regulation of HR repair by affecting the process of DNA-end resection through interacting CtIP. Interestingly, USP4 has no overt impact on the ubiquitination of CtIP despite their interaction, suggesting CtIP is not a substrate of USP4 DUB activity in this scenario^31,47^, and other potential substrates could exist. Identification of these DUB substrates is important for our understanding the role and mechanism of USP4 in the HR pathway.

Here, we identified USP4 as a crucial deubiquitinating enzyme that specifically regulates BRCA1 stability. USP4 physically interacts with and deubiquitinates BRCA1, thus saving BRCA1 from UPS-meditated ubiquitination and degradation. Depletion of USP4 significantly destabilizes BRCA1. Importantly, clinical mutations of USP4 display defective BRCA1 stability and impaired HR function. Besides, the expression of USP4 positively correlates with the BRCA1 level in breast tumor tissues. These findings suggest that USP4 may play an important role in the pathogenesis breast cancer.

## Results

### USP4 regulates BRCA1 stability

**Fig. 1:**
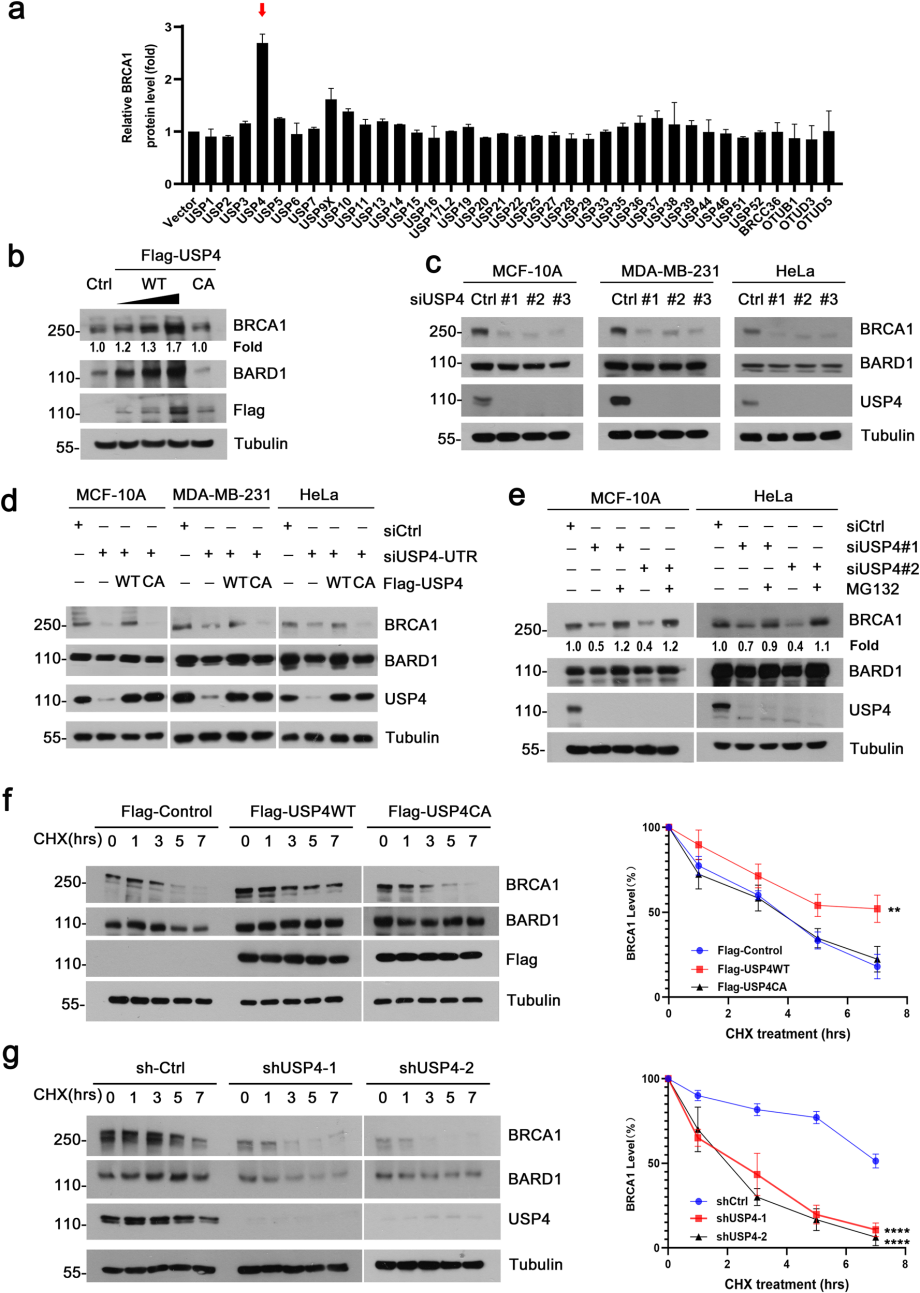
USP4 stabilizes BRCA1. **a** Screening for the deubiquitinating enzymes of BRCA1. HEK293T cells were transfected with the indicated DUB plasmids and lysed 48 h after transfection. Cell lysates were analyzed by Western blotting (WB). Each result was normalized to control vector. **b** Increasing amounts of Flag-USP4 WT or C311A mutant were transfected into HEK293T cells. Lysates were subjected to analysis by WB. **c** Depletion of USP4 by siRNAs in MCF-10A, MDA-MB-231 and HeLa cells and BRCA1 protein level was analyzed by WB. **d** MCF-10A, MDA-MB-231 and HeLa cells were transfected with control or siUSP4-UTR (siRNA against sequence at the untranslated regions of USP4 mRNA), 24 hours after transfection, USP4 WT or C311A mutant were introduced into cells for another 48 hours. BRCA1 protein level was analyzed by WB. **e** MCF-10A and HeLa cells were transfected with control or siRNAs against USP4. 72 hours after transfection, the cells were treated with MG132 (20 μM) for 4 h, then lysed and analyzed by WB using the indicated antibodies. **f** MCF-10A cells stably expressing control, Flag-tagged USP4 WT or C311A plasmids were treated with CHX (10 μg/ml) for the indicated time, and endogenous BRCA1 and ectopic USP4 were analyzed by WB. BRCA1 was quantified and normalized to Tubulin (right). Data were obtained from three independent experiments and presented as the mean ± SD. **p < 0.01, two-way ANOVA test. **g** MCF-10A cells stably expressing control, USP4 shRNAs were treated as in **f** for the indicated time, BRCA1 and USP4 were examined by WB. The level of BRCA1 was quantified and normalized to Tubulin (right). Data were obtained from three independent experiments and presented as the mean ± SD. ****p < 0.0001, two-way ANOVA test.

To investigate the role of DUBs in regulating BRCA1 stability, we transfected a panel of Flag or HA-tagged DUBs plasmids into HEK293T cells individually, and the expression of endogenous BRCA1 was examined by immunoblotting to screen for the potential DUBs of BRCA1. The results showed that overexpression of USP4 induced significant upregulation of BRCA1 protein, whereas other DUBs including USP9X had little effect (Fig. 1a and Supplementary Fig. 1a–d). This finding suggests that USP4 could be a major DUB for BRCA1. To confirm the result, we transfected HEK293T cells with either wild-type (WT) USP4 or its catalytic-inactive mutant C311A plasmid. As expected, forced expression of USP4 WT, but not the C311A mutant, resulted in accumulation of BRCA1 in a dose-dependent manner. This result indicates that the enzymatic activity of USP4 is required for BRCA1 stabilization (Fig. 1b). Consistent with the requirement of USP4 in maintaining the stability of BRCA1, depletion of endogenous USP4 by short interfering RNA (siRNA) resulted in a marked reduction in endogenous BRCA1 protein in MCF-10A cells (Fig. 1c, left panel). Similar results were observed in MDA-MB-231 and HeLa cells (Fig. 1c, middle and right panel). Importantly, the reduction of BRCA1 in USP4-siRNA depleted cells could be reversed by re-introduction of the siRNA-resistant wild-type USP4, but not the C311A mutant, indicating that USP4 deubiquitinating enzyme activity is critical for BRCA1 stability (Fig. 1d). On the other hand, the downregulation of BRCA1 in USP4-knockown cells can be effectively blocked by MG132 treatment (Fig. 1e), suggesting that USP4 probably regulates BRCA1 protein level in a proteasome pathway-dependent manner.

To further investigate the role of USP4 in regulating BRCA1 stability, we performed cycloheximide (CHX) chase assays in MCF-10A cells stably expressing ectopic USP4 or USP4 short hairpin RNA (shUSP4) (validated by Western blotting in Supplementary Fig. 2a). Overexpression of wild-type USP4 prolonged the half-life of BRCA1, whereas the catalytic-inactive mutant of USP4 (C311A) had no such effects (Fig. 1f). In contrast, shRNA-mediated depletion of USP4 resulted in a decrease in the stability of BRCA1 (Fig. 1g). Collectively, these data suggest that USP4 stabilizes BRCA1 through its deubiquitinating enzyme activity in cells.

### USP4 directly interacts with BRCA1, BARD1 and/or BRCA1/BARD1

**Fig. 2:**
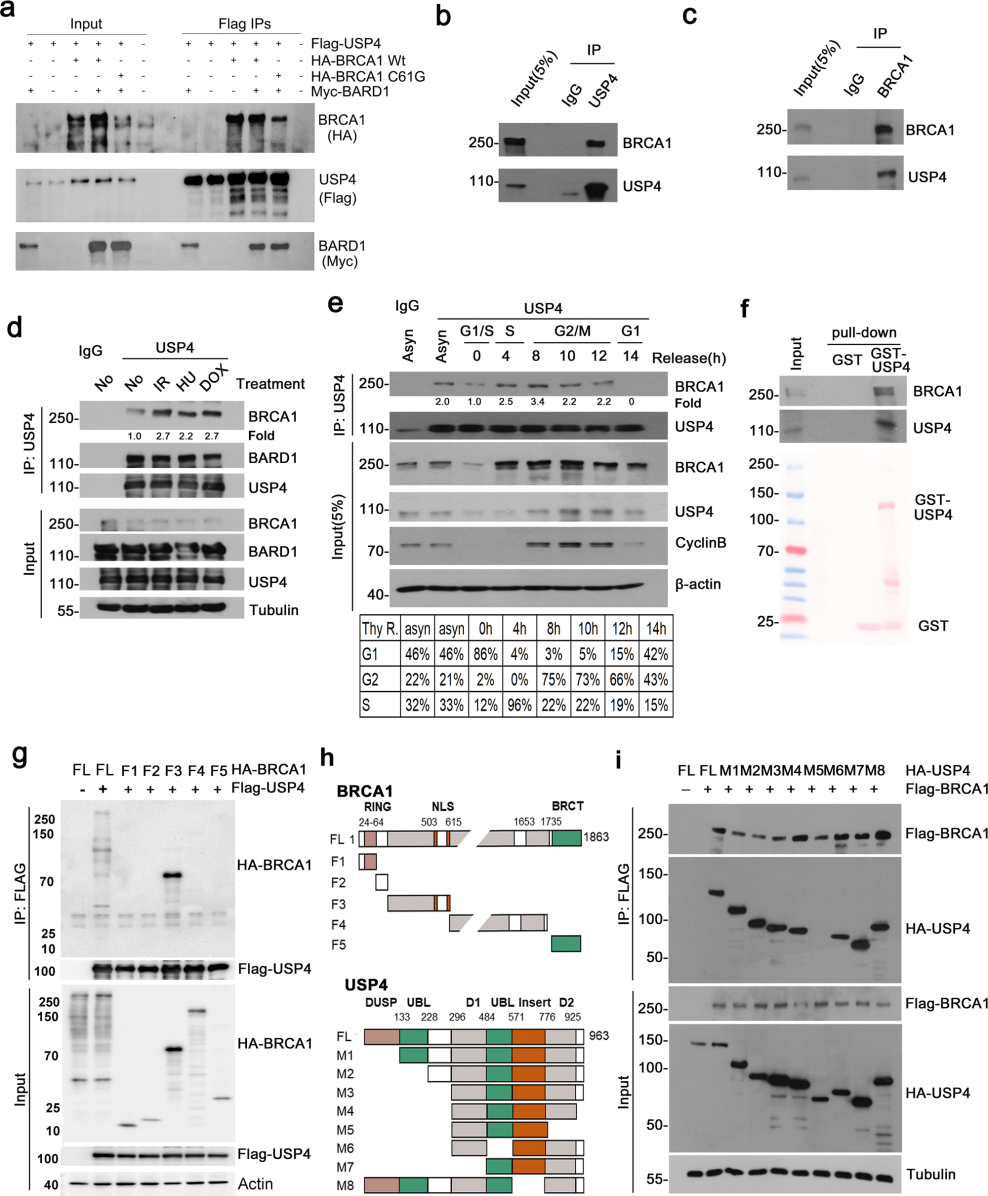
USP4 interacts with both BRCA1 and BARD1. **a** USP4 interacts with both BRCA1 and BARD1. 293 T cells were transfected with the indicated plasmids, and Flag-USP4 IPs were performed. BRCA1 and BARD1 were examined by WB using antibodies against HA or myc. **b** Endogenous USP4 interacts with BRCA1 in MCF-10A cells. Anti-USP4 antibody was used for IP and the blot was probed with anti-USP4 or BRCA1 antibody, respectively. **c** Endogenous BRCA1 interacts with USP4 in MCF-10A cells. Endogenous BRCA1 was pulled down by anti-BRCA1 antibody and the IPs was blotted with anti-BRCA1 or USP4 antibody, respectively. **d** Interaction of USP4 with BRCA1 upon treatment of DNA damage agent. HEK293T cells were treated with the indicated DNA damage agents and the IPs of USP4 were analyzed by WB with the indicated antibodies. BRCA1 pulled down by USP4 was quantified and normalized with that of control (No treatment). **e** Interaction between USP4 and BRCA1 during cell cycle. HeLa cells were synchronized to G1/S boundary by double-thymidine block, and then released for the indicated time. The cell lysates of G1, S and G2/M phase were subjected to immunoprecipitation using anti-USP4 antibody. The IPs were analyzed by WB with the indicated antibodies. BRCA1 pulled down by USP4 was quantified and normalized with that of G1/S. Cell cycle was analyzed by FACS, and the distribution of cell cycle was indicated. Asyn, asynchronous cells. **f** USP4 interacts with BRCA1. HEK293T cells transfected with BRCA1 were lysed and the lysates were incubated with either GST or GST-USP4. GST-pulldowns were blotted with the indicated antibodies. **g** HA-tagged full-length or truncated BRCA1 were co-expressed with Flag-tagged USP4 in HEK293T cells. Extracts were subjected to IP with anti-Flag-M2 antibody, and analyzed by WB. **h** Schematic diagram showing the constructs expressing full-length BRCA1 and its truncated fragments (Top panel), or the structure of USP4 and deletion constructs used (bottom panel). **i** HA-tagged USP4 constructs were co-expressed with Flag-tagged BRCA1 in HEK293T cells. Cellular extracts were subjected to IP with anti-Flag-M2 antibody, and analyzed by WB.

Most of the cellular BRCA1 form heterodimer with BRCA1-associated RING domain protein 1 (BARD1). To gain insights into the mechanism by which USP4 regulates BRCA1, we first examined whether USP4 interacts with BRCA1 and/or BARD1. 293 T cells were transfected with Flag-USP4, together with either BARD1 alone, BRCA1 alone, BARD1 plus BRCA1, or BARD1 plus BRCA1 C61G, a mutant of BRCA1 that does not interact with BARD1, and Flag IPs were performed. The result showed that BRCA1 were pulled down by Flag-USP4 from 293 T cells expressing either BRCA1 alone, or BRCA1 plus BARD1. Notably, BRCA1 C61G was also pulled down by Flag-USP4 (Fig. 2a). This result indicates that USP4 can interact with both BARD1-unbound BRCA1(“free” BRCA1) and may also bind BRCA1/BARD1 heterodimer. Similarly, BARD1 was pulled down by Flag-USP4 from cells expressing BARD1 alone, or BARD1 plus either BRCA1 Wt or C61G (Fig. 2a). Since BARD1 can only heterodimerize with BRCA1 Wt, but not its mutant C61G, these results indicate that USP4 can interact with BRCA1-unbound BARD1, or BARD1-unbound BRCA1, or BRCA1/BARD1. In the following study we mainly focused on the role of USP4 in the regulation of BRCA1. To confirm the interaction of USP4 with BRCA1, HEK293T cells were transfected with Flag-tagged USP4 WT or the C311A mutant plasmid and a Co-IP experiment was performed. The Co-IP assay indicated that endogenous BRCA1 could interact with USP4 in a manner that is independent of its DUB activity (Supplementary Fig. 2b).

To further confirm the in vivo interaction between USP4 and BRCA1, Co-IP of endogenous USP4 and BRCA1 from MCF-10A cells were performed and the results showed that USP4 was efficiently co-immunoprecipitated with BRCA1, and vice versa (Fig. 2b, c). Notably, USP4 was reported to interact with CtIP to promote DNA-end resection while had no effect on CtIP ubiquitination. CtIP is also known to interact with BRCA1 to participate HR repair. Based on this, we then examined whether USP4 and BRCA1 could interact with each other in the absence of CtIP. Indeed, endogenous USP4 could be immunoprecipitated with BRCA1 in cells depleted of CtIP (Supplementary Fig. 2c). This result suggests that USP4 can interact with BRCA1 in a manner independent of CtIP. Interestingly, the interaction between USP4 and BRCA1 was enhanced with IR/ doxorubicin treatment (Fig. 2d), suggesting their interactions might be DNA damage inducible.

BRCA1 protein fluctuates during cell cycle, with its level low at G1, increases from G1 through S phase, and peaks at G2/M phase. To investigate the physiological significance of USP4-BRCA1 association, we analyzed the protein level and interaction between BRCA1 and USP4 during cell cycle. HeLa cells were synchronized to G1/S phase by double-thymidine block, and each phase of cells were collected by thymidine release. Immunoprecipitation result showed that the interaction between USP4 and BRCA1 is almost undetectable at early G1, and increases from late G1 through S, and peaks at G2/M, which concomitantly correlates with the levels of BRCA1 at these phases (Fig. 2e). This result is consistent with a role of USP4 in stabilizing BRCA1, suggesting that USP4 physiologically interacts with BRCA1 to control BRCA1 protein level during the progression of cell cycle.

To confirm the interaction between USP4 and BRCA1, we performed binding assay using recombinant USP4 fused with GST (GST-USP4). The results showed that the purified GST-USP4 but not the GST control was able to pull-down BRCA1 in vitro (Fig. 2f), suggesting that a direct interaction might exist between USP4 and BRCA1. To map the USP4-binding region on BRCA1, we generated protein truncations from cells expressing different BRCA1 fragments and several USP4 deletion mutants. First, we co-expressed Flag-tagged USP4 along with a series of truncations of HA-tagged BRCA1, co-immunoprecipitation assays demonstrated that BRCA1-F3 (168–615aa) was required for its binding to USP4 (Fig. 2g, h). We next mapped the region of USP4 required for its interaction with BRCA1, and the result showed that D2 domain of USP4 was responsible for its binding to BRCA1 (Fig. 2h, i).

### USP4 deubiquitinates BRCA1

**Fig. 3:**
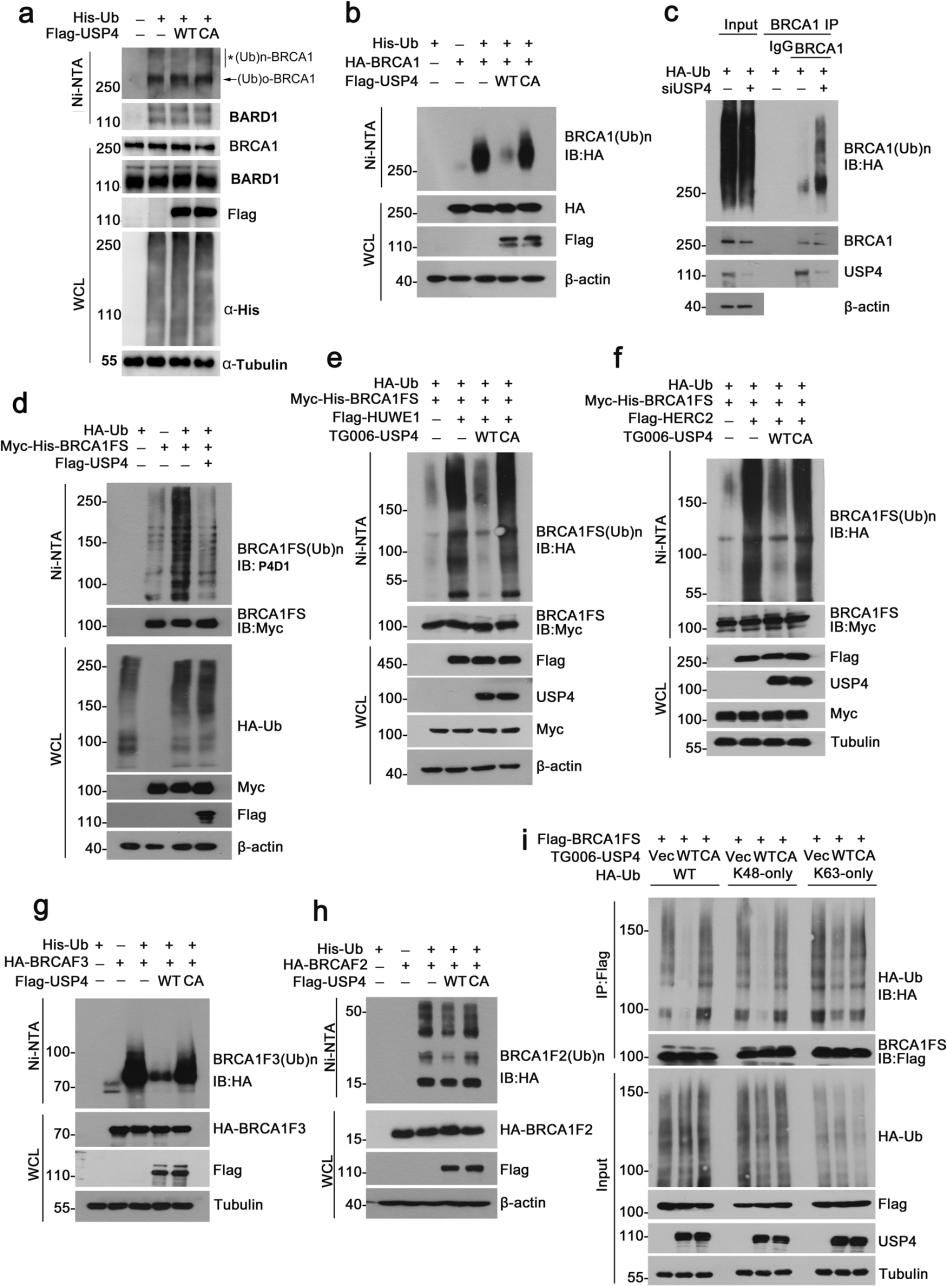
USP4 deubiquitylates BRCA1. **a** USP4 WT, but not its CA mutant, deubiquitinates BRCA1. HEK293T cells transfected with the indicated plasmids were treated with MG132 for 4 h before harvest. Ubiquitinated species was pulled down using Ni-NTA and BRCA1 ubiquitination was analyzed by WB. WCL, whole cell lysate; *, polyubiquitinated BRCA1 species; arrow, mono or oligo-ubiquitinated BRCA1. **b** HEK293T cells transfected with the indicated constructs were treated as in **a**, and the ubiquitinated species were pulled down using Ni-NTA and BRCA1 ubiquitination was analyzed by WB. **c** Depletion of USP4 promotes BRCA1 ubiquitination. MCF-10A cells transfected with HA-Ub were depleted of USP4 by siRNA. Cells were then treated with MG132 for 4 h before harvest. BRCA1 was immunoprecipitated and its ubiquitination was analyzed by WB with anti-HA antibody. **d** HEK293T cells were transfected with the indicated plasmids and BRCA1 were pulled down by Ni-NTA and ubiquitination was analyzed by WB. **e** The ubiquitylation of BRCA1FS was performed in HEK293T cells transfected with the reported BRCA1 E3 ligase HUWE1 together with USP4 WT or CA mutant. **f** BRCA1FS ubiquitylation was assessed in HEK293T cells transfected with the reported BRCA1 E3 ligase HERC2 together with USP4 WT or CA mutant. **g**, **h** The ubiquitylation of BRCA1 truncates was assessed in HEK293T cells transfected with BRCA1-F3 (**g**) or BRCA1-F2 (**h**) together with USP4 WT or CA mutant. **i** USP4 deubiquitinates mainly the K48-linked ployubiquitin chains on BRCA1. HEK293T cells transfected with BRCA1FS, USP4, and ubiquitin WT, K48-only or K63-only plasmids were subjected to Ni-NTA pull-down and analyzed by WB to detect the ubiquitylated form of BRCA1FS.

Since USP4 is a DUB, together with the fact that the reduction of BRCA1 after USP4 depletion was restored after MG132 treatment, we propose that USP4 may regulate BRCA1 through deubiquitylation, thus antagonizing BRCA1 ubiquitination by its E3 ligases. Indeed, ectopic expression of USP4 WT, but not the C311A mutant, which is still capable of interacting with BRCA1, reduced both endogenous BRCA1 ubiquitylation (Fig. 3a, mostly polyubiquitinated BRCA1 species at high MW) and exogenous-overexpressed BRCA1 ubiquitylation in cells (Fig. 3b), suggesting that the DUB catalytic activity of USP4 is critical for USP4-dependent deubiquitylation of BRCA1. Conversely, silencing USP4 expression increased endogenous BRCA1 ubiquitylation level in cells after MG132 treatment (Fig. 3c). Given that full-length BRCA1 is a protein with high molecular weight, to better visualize its ubiquitination, we employed BRCA1FS (65–772 amino acid), a truncated form of BRCA1 without the RING domain, as substrate in ubiquitination assays. Previous studies have demonstrated that BRCA1FS could be ubiquitinated by BRCA1’s specific E3 ligases such as HERC2, HUWE1 and CRL4-DCAF8L1^19,21,22^. As shown, overexpression of Flag-tagged USP4 dramatically decreased the polyubiquitination of Myc-His-tagged BRCA1FS (Fig. 3d), again indicating that USP4 deubiquitinates BRCA1.To investigate whether USP4 could antagonize the activities of HERC2 and HUWE1 in ubiquitinating BRCA1, we introduced HUWE1 or HERC2 into HEK293T cells, and compared the ubiquitination of BRCA1FS in the absence or presence of USP4 WT and C311A mutant. As expected, expression of HERC2 or HUWE1 induced extensive ubiquitination of BRCA1FS, and BRCA1FS ubiquitination was then suppressed by USP4 WT, but not the C311A mutant (Fig. 3e, f). Similar results were obtained when assessing ubiquitination of other BRCA1 truncations such as F2 and F3 (Fig. 3g, h, Supplementary Fig. 3), which dominated the interaction between USP4 and BRCA1.

Previous studies have shown that USP4 can cleave both K48 and K63-linked polyubiquitin chains^48,49^. To determine the type of ubiquitin chains USP4 cleave on BRCA1, we performed in vivo deubiquitylation assays utilizing ubiquitin mutant K48 (K48-only) or K63 (K63-only). The result suggested that K48-linked ubiquitin chains of BRCA1 are the major forms of ubiquitin linkages hydrolyzed by USP4 (Fig. 3i). Together, these data demonstrated that USP4 targets BRCA1 for deubiquitylation, and counteracts HERC2 and HUWE1-mediated K48-linked polyubiquitination of BRCA1, indicating that USP4 is a bona fide DUB of BRCA1.

### USP4 regulates the cellular functions of BRCA1 in DNA damage repair

**Fig. 4:**
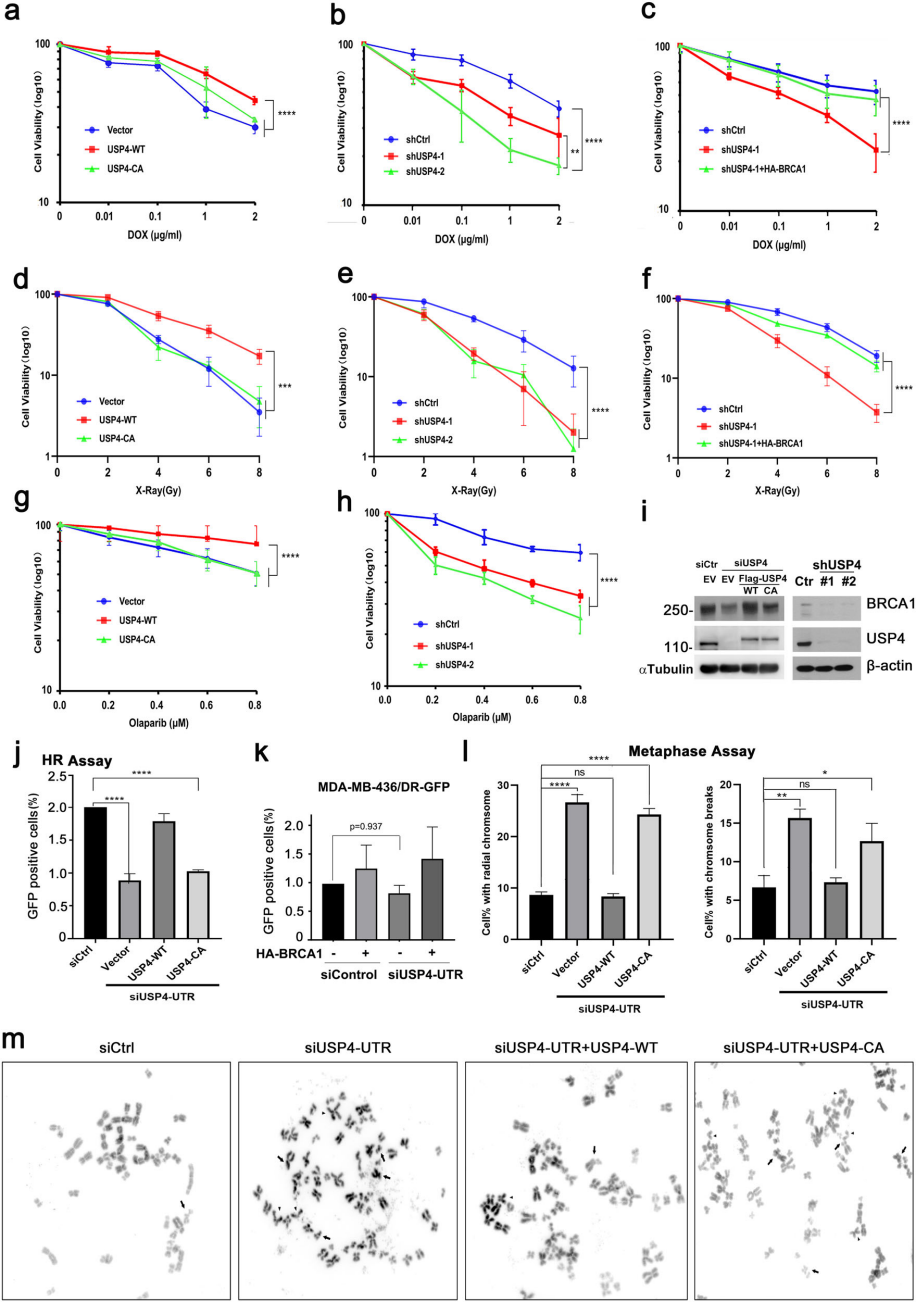
USP4 regulates BRCA1-mediated DNA damage repair. **a**, **b** Cell sensitivity of MDA-MB-231 cells stably expressing USP4 WT or CA mutant (**a**) or USP4 shRNAs (**b**) when treated with doxorubicin. **c** The sensitivity to doxorubicin with MDA-MB-231 cells depleted of USP4 when transfected with HA-BRCA1 or control plasmids. **d**, **e** Cell sensitivity of MDA-MB-231 cells stably expressing USP4 WT or CA mutant (**d**) USP4 shRNAs (**e**) after X-ray treatment. **f** The cell sensitivity of MDA-MB-231 cells depleted of USP4 when transfected with HA-BRCA1 plasmids after X-ray treatment. **g**, **h** The cell sensitivity of MDA-MB-231 cells stably expressing USP4 WT or CA mutant (**g**) or USP4 shRNAs (**h**) when treated with PARP inhibitor. **i** WB validation of the depletion of USP4 in MDA-MB-231 or shUSP4 stable cell lines used for function analysis. Cell survival rate was assessed by CCK8 assay, and the data were from three independent experiments and presented as the mean ± SD. **j** U2OS DR-GFP cells were depleted of USP4 using siUSP4-UTR and reconstituted with the indicated constructs. HR efficiency was determined. Data were obtained from three independent experiments and presented as the mean ± SD. ****p < 0.0001, Student’s *t* test. **k** USP4 was depleted with siUSP4-UTR in MDA-MB-436 cells stably integrated with repeat green fluorescent protein (DR-GFP) cassette. HR efficiency of cells reconstituted with the indicated constructs were assessed. Data were presented as the mean ± SD of three independent experiments. p > 0.05, Ordinary one-way ANOVA. **l**, **m** Chromosome aberrations in HeLa cells transfected with the indicated constructs. Metaphase spreads (**m**) were analyzed. Arrowhead indicates chromosome breaks, triangle indicates radial chromosomes. The indicated aberrations (**l**) are quantified and presented as the mean of three independent experiments, 100 metaphases per experiment were counted. Student’s *t* test, * *p* < 0.05, ** *p* < 0.01, ****p < 0.0001.

Since BRCA1 plays a key role in homologous recombination and cell cycle checkpoint^50^, and USP4 was reported to influence HR repair through recruiting CtIP to the sites of DNA damage^31,47^, we therefore believe that USP4 may participate DNA damage repair through an alternative mechanism by which it targets BRCA1. To investigate the role of USP4 in BRCA1-mediated DNA damage repair, we assessed the sensitivity of control cells and cells depleting or overexpressing USP4 in response to radiation or treatment of DNA-damaging agents. MDA-MB-231 cells stably expressing or depleting USP4 were treated with antitumor drug doxorubicin. As expected, overexpression of USP4 WT, but not the CA mutant conferred cells resistance to the DNA damage agents (Fig. 4a), whereas depletion of USP4 by two individual shRNAs sensitized cells to doxorubicin treatment (Fig. 4b).

To verify the functional association between USP4 and BRCA1 in this process, we reintroduced BRCA1 in MDA-MB-231 cells stably depleted of USP4, and cells were then exposed to doxorubicin treatment. The results further confirmed that re-expression of BRCA1 was capable of restoring the cellular sensitivity phenotype caused by USP4 knockdown (Fig. 4c). Similar results were obtained when cells were treated with X-ray, etoposide (Fig. 4d–f and Supplementary Fig. 4a–c), or Olaparib (Fig. 4g, h), suggesting that USP4 regulates the cellular response to DNA-damaging agents in a BRCA1-dependent manner. Additionally, we found that USP4 deficiency resulted in a decrease in HR efficiency using integrated reporter assays for HR in U2OS cells, which was restored by reconstitution with USP4 WT, but not the CA mutant (Fig. 4j and Supplementary Fig. 4d). However, this regulatory role of USP4 on HR efficiency was not observed in MDA-MB-436 cells that are defective of BRCA1 (Fig. 4k). Consequently, knockdown of USP4 raised the DNA damage-induced chromosome aberrations including radial chromosomes and chromosome breaks, and reconstituting these cells with USP4 WT, but not the USP4-C311A mutant, rescued the chromosome phenotypes (Fig. 4l, m). Taken together, these results support the idea that USP4 plays an essential role in BRCA1-mediated HR repair through a mechanism by regulating BRCA1 stability in addition to its role in CtIP-mediated DNA-end resection.

### Clinical USP4 mutations from human breast cancer are defective in deubiquitinating and stabilizing BRCA1

**Fig. 5:**
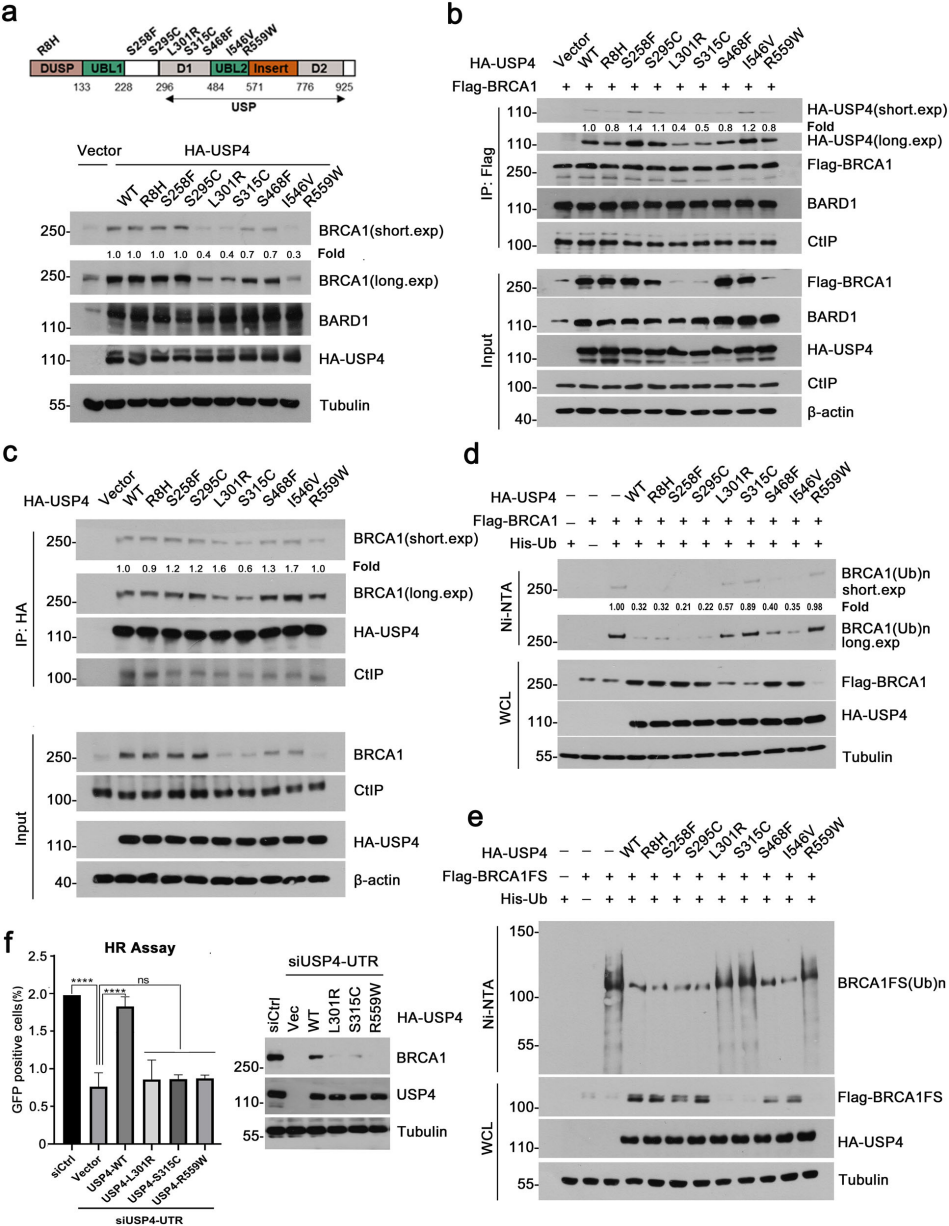
Identification and characterization of USP4 loss-of-function mutations in human cancers. **a** Expression of USP4 mutants caused reduction of BRCA1. Schematic diagram of USP4 structure and location of point-mutation (upper panel); Mutations of USP4, which were reported in cBioPortal database were selected for generating recombinant plasmids and introduced into HEK293T cells. Endogenous BRCA1 were analyzed by WB. Wild-type USP4 was used as positive control (lower panel). The expression of BRCA1 in cells transfected with USP4 was quantified and normalized with that in control (USP4 WT). **b** Interaction of BRCA1 with USP4 mutants. HA-tagged USP4 mutants from breast cancers were co-expressed with Flag-tagged BRCA1 in HEK293T cells. Flag-BRCA1 was immunoprecipitated and blotted with the indicated antibodies. USP4 WT or each mutant protein pulled down by Flag-BRCA1 was quantified and normalized with USP4 WT. **c** Interaction of USP4 mutants with BRCA1. HA-tagged USP4 mutants were overexpressed in HEK293T cells. HA-USP4 WT and mutants were immunoprecipitated and analyzed with the indicated antibodies. BRCA1 pulled down by USP4 WT or each mutant was quantified and normalized with that by USP4 WT. **d** Clinic USP4 mutations are defective in deubiquitinating BRCA1. HA-tagged USP4 mutants from breast cancers were co-expressed with Flag-tagged full-length BRCA1 and His-Ub in HEK293T cells. Ubiquitinated proteins were pulled down using Ni-NTA, and blotted with BRCA1 antibody. **e** Clinic USP4 mutations are defective in deubiquitinating BRCA1. HA-tagged USP4 mutants from breast cancers were co-expressed with Flag-tagged BRCA1FS and His-Ub in HEK293T cells. Ubiquitinated proteins were pulled down using Ni-NTA, and blotted with anti-Flag antibody. **f** Mutation of USP4 induced a reduction in HR efficiency. U2OS DR-GFP cells were depleted of endogenous USP4 with siUSP4-UTR and then reconstituted with USP4 WT, or mutants (L301R, S315C, or R559W). HR efficiency was assessed. Data were obtained from three independent experiments and presented as the mean ± SD. Left, HR efficiency normalized to control, ****p < 0.0001; Right, WB conformation of transfection.

BRCA1 plays a pivotal role in the suppression of human breast cancer. Approximately 50% of familial breast cancers harbor BRCA1 mutations, and a reduction in the levels of BRCA1 protein is also frequently observed in many sporadic breast cancers^1,51^. Based on its role in stabilizing BRCA1, we speculate that USP4 mutations could exist and be a mechanism for the downregulation of BRCA1 in those breast cancers. Therefore, we searched TCGA database and a series of USP4 mutations were found in gynecologic cancers including breast cancer, ovarian cancer and cervical cancer. Based on these clinical mutations identified from patients, we then constructed a total of 46 point-mutation of USP4 plasmids, 26 of which are located in the bipartite catalytic domain (also known as USP domain) of USP4. In order to define which mutation is associated with BRCA1, we introduced these USP4 mutants into HEK293T cells and examined their impact on BRCA1 protein level. Strikingly, three USP4 mutants from breast cancer, namely L301R, S315C, and R559W, failed to upregulate endogenous BRCA1 protein level (Fig. 5a and Supplementary Fig. 5a), whereas most of other mutants could still efficiently promote the accumulation of BRCA1 like USP4 WT (Supplementary Fig. 5b–d). Consistent with this, CHX chase assays revealed that these three mutants failed to prolong the half-life of BRCA1 (Supplementary Fig. 5e–g).

To better understand the mechanism underlying the loss-of-function of these three mutants, we first investigated whether these mutants could still interact with BRCA1. HEK293T cells were co-transfected with Flag-tagged BRCA1 and HA-tagged USP4 WT or mutants, and Co-IP experiments were performed. The results revealed that the interaction of BRCA1 with L310R or S315C mutant was remarkably compromised, whereas BRCA1’s interaction with R559W was slightly decreased. Similar results were obtained in the reciprocal IP of HA-tagged USP4 and endogenous BRCA1 (Fig. 5b, c). These results suggest that USP4 mutations in the D1 domain mainly affect their binding to BRCA1. Interestingly, like L310R and S315C, R559W mutant still can destabilize BRCA1 despite of its ability to interact with BRCA1.

Next, we asked whether these USP4 mutants could deubiquitinate BRCA1. HEK293T cells were co-transfected with USP4 WT or mutants together with Flag-tagged full-length BRCA1 or BRCA1FS truncation, and ubiquitination of BRCA1 was assessed. As expected, USP4 WT efficiently deubiquitinated BRCA1. Surprisingly, L301R, S315C, and R559W mutants were completely defective in deubiquitinating both BRCA1 full-length (Fig. 5d) and BRCA1FS truncation (Fig. 5e), while other USP4 mutants had little or no effect in doing so. Consistent with their inability to stabilize BRCA1, L301R, S315C, and R559W mutants were unable to restore HR efficiency as USP4 WT in cells depleted of endogenous USP4 (Fig. 5f). Taken together, these results suggest that mutations of USP4 L301R, S315C, and R559W may play a role in the pathogenesis of breast cancer, probably through destabilizing BRCA1.

### USP4 is downregulated in human breast cancers and positively correlated with BRCA1 protein level in breast cancer patients

**Fig. 6:**
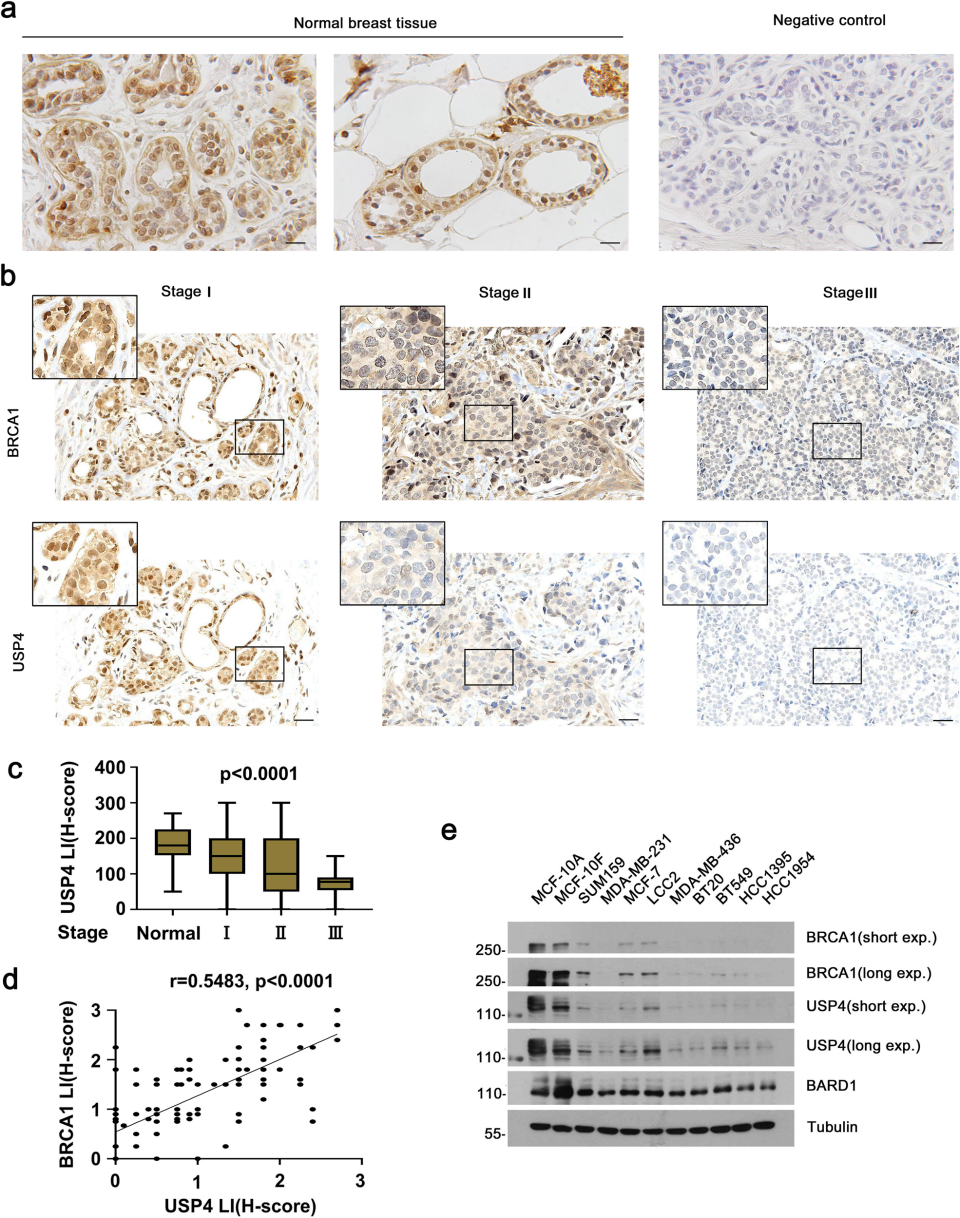
USP4 protein is downregulated in human breast cancer and positively correlates with BRCA1. **a** IHC staining of USP4 in normal human breast tissues. Breast lobules (left) and ducts(middle) were shown; negative control for IHC staining (right). Scale bar, 20 μm. **b** Correlation between BRCA1 and USP4 expression was compared. 90 cases of breast cancer tissues within different clinicopathological TNM stages were analyzed by IHC. Representative photographs of BRCA1 and USP4 immunoreactivity in breast cancers were shown. Normal adjacent breast tissues and paired cancer tissues with different TNM stage in a breast tissue microarray are shown in supplementary Fig. 6. Scale bar, 200 μm. **c** USP4 and BRCA1 protein levels in breast cancer tissues of different stages were quantified and presented as boxplots. Within each box, horizontal black lines denote median values, boxplots represent the interquartile range (25th to 75th percentile) and whiskers extend to the minimum and maximum values within 1.5 times the interquartile range. Data were analyzed by the *χ*^2^ test. **d** Correlation analysis of USP4 and BRCA1 in breast cancer tissues. The *χ*^2^ test was used for statistical analysis. Correlation coefficient, *r* = 0.5483, *p* < 0.0001. **e** Expression of USP4 and BRCA1 in human breast cancer cell lines. Breast cancer cell lines were analyzed by WB.

To determine the relevance of regulation of BRCA1 by USP4 in patients, we first performed immunohistochemical (IHC) analysis to examine USP4 expression in normal human breast tissues (8 cases). USP4 staining was detected in the epithelial cells of breast lobules and ducts, mainly luminal epithelial cells. High expression was seen in the nuclei of approximate half of the luminal epithelial cells (Fig. 6a). USP4 expression was also observed in stromal cells. This finding suggests that USP4 might play a role in the differentiation and development of mammary gland. We next examined a breast tissue microarray, including 90 cases of breast tumor samples paired with adjacent normal tissues. Representative images as shown (Fig. 6b and Supplementary Fig. 6a), USP4 expression was significantly downregulated in the breast cancer tissues compared with human normal breast tissues and the corresponding adjacent tissues. Notably, USP4 protein level was correlated with tumor grade in patients with invasive breast cancer. High USP4 expression was observed in low-grade cancers, and vice versa, indicating that lower USP4 expression might correlate with a poorer differentiation status and more aggressive phenotype in breast cancer patients. To further validate this finding, we analyzed the correlation between USP4 expression and the tumor node metastasis (TNM) stage in a larger-scale array of 226 breast cancer tissue samples. The result demonstrated that the expression of USP4 was much lower in stage III cases than that in other stages, suggesting a negative relationship between USP4 expression and breast cancer TNM stage (Fig. 6c). Furthermore, we assessed USP4 expression and its correlation with BRCA1 in consecutive tissue sections of a total of 226 breast carcinoma samples in various types mentioned above. Decreased BRCA1 and USP4 was observed in 59% (134 of 226) and 63% (143 of 226) of breast cancer respectively, whereas only 10% (23 of 226) and 8% (18 of 226) exhibited high expression (Table 1), suggesting that both BRCA1 and USP4 are downregulated in human breast cancers. Besides, a positive correlation between USP4 and BRCA1 protein levels was also observed (Fig. 6d). Consistently, Western blot analysis of USP4 and BRCA1 in multiple breast cancer cell lines including luminal and triple negative breast cancers, and normal human mammary epithelial cells further implied that expression of USP4 and BRCA1 is positively correlated (Fig. 6e).

**Table 1:** Correlation of USP4 and BRCA1 expression in breast cancer

USP4 protein level	BRCA1 protein level				*P* value
	−/+	++	+++	Total	
−/+	102 (45.1)	33 (14.6)	8 (3.5)	143 (63.2)	*p* < 0.0001
++	28 (12.4)	30 (13.3)	7 (3.1)	65 (28.8)	
+++	4 (1.8)	6 (2.7)	8 (3.5)	18 (8.0)	

We investigated cancer-associated alterations of USP4 in cBioPortal database (https://www.cbioportal.org) to explore whether USP4 low expression or genomic mutation is linked with breast cancer, as well as other gynecologic cancers. USP4 alterations in breast, ovarian, cervical and endometrial cancers from 49 studies (18486 samples) were analyzed. The result showed that USP4 depletion and mutation were frequently observed in these gynecologic cancers (Supplementary Fig. 6b).

Collectively, these findings indicate that USP4-facilitated BRCA1 stabilization potentially plays a tumor-suppressive role in breast carcinogenesis.

## Discussion

The homeostasis of a protein is crucial for the proper execution of its biological function, and is regulated by multiple mechanisms, dysregulation of this process would led to alteration in the quantities of cellular proteins, which is a common cause for many diseases including cancer.

BRCA1 is a key player in DDR pathway. Loss or decreased expression of BRCA1 is reported in 30–60% breast cancer tissues^5,52,53^. Cellular BRCA1 protein levels are sensitive to MG132 treatment. This phenomenon strongly suggests that the ubiquitination and subsequent UPS-dependent degradation play an important role in the control of BRCA1 stability. Indeed, growing evidence have shown that multiple E3 ligases including HERC2, FBXO44, HUWE1, and CRL4-DCAF8L1 are involved in the ubiquitination of BRCA1^19–21,54^. Since ubiquitination can be reversed by DUBs, it is therefore that DUBs are also key factors for regulating BRCA1 stability and DNA repair.

In this study we demonstrate that USP4 is an essential regulator of BRCA1 acting as a bona fide DUB to maintain BRCA1 stability. Both BRCA1 and USP4 are mainly localized in the nucleus, and their association is cell cycle-related, which peaks in S and G2 phases^43,55^. This result suggests a physiological role of USP4 in regulating the homeostasis of cellular BRCA1 protein during cell cycle progression.

The role of USP4 in HR-directed DNA damage repair has been well documented. Previous studies showed that it promotes homologous recombination through interacting with CtIP/MRN and regulating CtIP recruitment to initiate DNA-end resection^31,47^. However, USP4 showed no effect on CtIP ubiquitination, suggesting USP4 contributes to DNA damage repair through a mechanism that is independent of its DUB activity. Here we provide an alternative mechanism by which USP4 regulates DNA damage repair through regulating BRCA1 stability and abundance. We demonstrate that USP4 act as BRCA1 deubiquitinating enzyme to antagonize BRCA1 ubiquitination. Notably, this USP4-mediated de-ubiquitination of BRCA1 is CtIP independent, indicating that USP4-BRCA1 axis is a distinct pathway other than USP4-CtIP in regulating HR.

BRCA1 plays critical role in the suppression of breast cancer. Loss or reduction of BRCA1 protein is often observed not only in hereditary but also in sporadic breast cancers^5,52,53^. Reduction of BRCA1 protein or aberrant localization of BRCA1 have been reported in non-familial breast and ovarian cancers^4,5,56^, the underlying mechanism for the destabilization of BRCA1 in this context is unclear. Based on the role of USP4 in stabilizing BRCA1, we infer that loss or aberrant expression of USP4 may play a role in non-familiar breast and ovarian cancer and could correlate with the altered BRCA1 level. Consistent with our assumption, we observed decreased USP4 expression in 63% of the breast cancer tissues, and a positive correlation between USP4 and BRCA1 was found (Table 1). In addition, we identified several loss-of-function mutations of USP4 from breast and ovarian cancer database. Importantly, we further demonstrate that USP4 mutants (L301R, S315C and R559W) that are defective either in DUB activity or in interacting with BRCA1 can cause significant decrease in BRCA1 protein level and hence confer increased sensitivity to IR or anticancer drugs such as DOX, ETO or PARPi. Interestingly, many of these USP4 loss-of-function mutations, including L301R, S315C and R559W, are found mainly located in the catalytic domain of USP4, suggesting that the DUB activity is critical for maintaining BRCA1 stability and hence its tumor suppression function. We also identified several USP4 mutants (for example R8H) whose mutation can lead to the increased USP4 protein stability, but still can cause a significant reduction of BRCA1 when overexpressed. This phenomenon suggests that these mutation types have a dominant negative effect, and BRCA1 could still be downregulated in the breast cancers bearing these USP4 mutations. This finding might provide an additional explanation for the high expression of USP4 in 8–10% of breast cancer observed in this study. Further studies are needed to address whether BRCA1 is lowered and whether USP4 is mutated in these USP4 high expression cases. Taken together, our study suggest USP4 is a key regulator of BRCA1 and dysregulation could play an important role in the pathogenesis of breast cancer. Since breast cancers with defective BRCA1 and HR pathway are sensitive to DNA damage-inducing anticancer drugs such as PARPi, our study provides a novel target for the treatment of breast cancer, as well as the development of anticancer drugs.

## Methods

### Antibodies

Primary antibodies used in this study were as follows: mouse anti-USP4 (66822; Proteintech1:1000 dilution for WB, 1:300 dilution for IHC); mouse anti-BRCA1 (sc-6954; Santa Cruz; 1:300 dilution for WB); rabbit anti-BRCA1(ab16780; Abcam;1:400 dilution for IHC); rabbit anti-BRCA1 (ab191042; Abcam;1:1000 dilution for WB); rabbit anti-BARD1 (NB100; Novus Biologicals; 1:1000 dilution for WB); mouse anti-BARD1 (sc-74559; Santa Cruz; 1:500 dilution for WB); mouse anti-Flag (F3165; Sigma; 1:5000 dilution for WB); rabbit anti-HA (3924 S; Cell Signaling Technology; 1:1000 dilution for WB); mouse anti-Myc (M4439; Sigma;1:5000 dilution for WB); mouse anti-ubiquitin (3936 S; Cell Signaling Technology; 1:1000 dilution for WB); mouse anti-α-His (2366 S; Cell Signaling Technology; 1:1000 dilution for WB); mouse anti-α-Tubulin (3873 S; Cell Signaling Technology; 1:3000 dilution for WB), rabbit anti-β-actin (AC026; ABclonal; 1:1000 dilution for WB); rabbit anti-GAPDH(5174 S; Cell Signaling Technology; 1:5000 dilution for WB). For WB, western blots were detected and analyzed using ChemiDoc Imaging system (Bio-Rad). The original WB blots were included in the supplementary information.

### Plasmid construction

Full-length and truncated USP4 gene were cloned into p3xFlag-CMV-10 or pCMV-HA to generate various plasmids for the expression of USP4 proteins; full-length USP4 mutants including C311A, R8H, S258F, S295C, L301R, S315C, S468F, I546V, R559W, R8C, F42C, K186N, D428Y, L459V, E463K, P480S, R501T, Q667E, I782T, G4E, R10Q, S72F, E166D, Q227H, P384S, V538L, S598L,Y822L, A860S, S39Q, R411Q, D517N, V572M, P591Q, Q769K, D780Y, A808Y, A869T, Q921K were generated using site-directed mutagenesis kit. For bacterial expression, GST-USP4 fusion protein plasmid was generated by subcloning the coding sequence of USP4 into the pGEX-6P-3 vector. Full-length and truncated BRCA1 genes were cloned into p3xFlag-CMV-10 or pCMV6-AC-3xHA or pcDNA4-Myc-His to generate plasmids for the expression of BRCA1 proteins.

### Cell culture and transfection

All cell lines used in this study were obtained from the American Type Culture Collection (ATCC). HEK293T and HeLa cells were cultured in DMEM (Macgene) with 10% FBS (Hyclone). MCF-7 cells were cultured in DMEM with 10% FBS and 0.01 mg/ml human recombinant insulin (Sigma, I9278). MDA-MB231 cells were cultured in Leibovitz’s L-15 with 10% FBS at 37 °C without CO2. MCF-10A and MCF-10F cells were cultured in DMEM/Ham’s F12 Medium (v/v = 1:1) with 5% horse serum, 20 ng/mL epidermal growth factor (Sigma, E9644), 0.01 mg/mL human insulin (Sigma, I9278), 100 ng/ml cholera toxin (Sigma, C8052), and 500 ng/mL hydrocortisone (Sigma, 614157). All cell lines were cultured at 37 °C with 5% CO2 except MDA-MB231. Transfection were performed using jetPRIME® (Thermofisher) or Lipofectamine 2000 (Invitrogen) according to manufacturer’s instructions.

### RNAi sequence

For siRNA transfection, cells were transfected using jetPRIME® (Thermofisher) following the manufacturer’s instructions. For lentiviral infection, shRNA lentiviral particles were packaged and transduced into the indicated cells in the presence of Polybrene. The sequences of USP4 siRNAs were as following: siUSP4-1, GGCAGACCUUGCAGUCAAATT; siUSP4-2, CCUACGAGCAGUUGAGCAATT; siUSP4-3, CGAGGCGUGGAAUAAACUATT; siUSP4-4(siUSP4-UTR) UUAAACAGG UGGUGAGAAATT. The sequences of USP4 shRNAs were as following: shUSP4-1, TTAAACAGGTGGUGAGAAA; shUSP4-2, CGAAGAATGGAGAGGAACA. The sequence of CtIP siRNA was GCUAAAACAGGAACGAAUCT T.

### Co-immunoprecipitation

Cells were collected and lysed on ice for 30 minutes in NETENG-400 buffer containing 400 mM NaCl, 20 mM Tris-HCl, pH 7.4, 0.5 mM EDTA, 1.5 mM MgCl_2_, 0.1% NP-40, and 10% Glycerol, with protease inhibitors and phosphatase inhibitors (Sigma and Selleck). The samples were centrifuged at 12,000 rpm for 10 min, and the supernatants were diluted with NETENG-0 (containing all the same ingredients as NETENG-400 except for without NaCl) by adding 1.67 volume of the supernatant. For immunoprecipitation, the lysates were incubated with 2 μg BRCA1 antibody (Santa Cruz, sc-6954) or 1 μg USP4 antibody (Proteintech, 66822) or normal mouse IgG overnight at 4 °C with rotation, and protein-A/G agaroses (Roche) were then added, and incubated for an additional 2–4 h. For tagged protein immunoprecipitation, protein samples and anti-Flag-M2 or Anti-HA Agarose (Sigma-Aldrich) were incubated at 4 °C with rotation for 6 h. The immunocomplexes were then washed with NETENG-150 buffer (containing the same ingredients as NETENG-400 except for with 150 mM NaCl). Both the lysates and eluates were examined by WB with the indicated antibodies.

### Ni-NTA pull-down

48 h after transfection, HEK293T cells were harvested and lysed on ice for 30 minutes in Buffer B (8 M Urea, 100 mM NaH_2_PO_4_, 10 mM Tris-Cl pH 8.0, and 25 mM imidazole) containing protease and protein phosphatase inhibitors. The lysates were then subjected to ultrasonication for 30 s (MW 30 W), and clarified by centrifugation, followed by incubation with Ni-NTA agarose (QIAGEN) for 4 h. The beads were washed four times with Buffer C (8 M Urea, 100 mM NaH_2_PO_4_, 10 mM Tris-Cl pH 6.3, 25 mM imidazole), and eluted with Buffer E (8 M Urea, 100 mM NaH_2_PO_4_, 10 mM Tris-Cl pH 4.0, 250 mM imidazole). The eluants were examined by WB with the indicated antibodies.

### In vivo ubiquitylation

For BRCA1 in vivo ubiquitylation, MCF-10A cells were first transfected with either control or USP4-siRNA oligos and re-plated in 100-mm dishes 24 h after transfection. Cells were transfected again 24 h later with HA-ubiquitin, and treated with 20 μM MG132 for 4 h. Forty-eight hours later, cells were lysed in NETENG-400 containing protease and protein phosphatase inhibitors. The lysates were incubated with anti-BRCA1 antibody (Santa Cruz, sc-6954) for 3 h, followed by incubation with protein-A/G agarose for additional 8 h at 4 °C. The immnunoprecipitates were examined by WB with the indicated antibodies.

### GST pull-down

Recombinant protein GST-USP4 was expressed in E. coli Rosetta with 1 μM IPTG treatment at 16 °C for 16 h, and purified using glutathione-Sepharose 4B beads. The purified GST-fusion protein was incubated with the cell lysates of HEK293T cells expressing BRCA1 overnight at 4 °C, and the beads were washed with PBS and eluted. The eluants were examined by WB with the indicated antibodies.

### In vitro proliferation assay

MCF-10A cells stably expressing Flag-tagged USP4 WT or CA mutant, shCtrl (Control), shUSP4-1, shUSP4-2 were seeded in 96-well plates at a density of 10^3^ cells/well, and treated with DOX, IR, ETO or Olaparib (Selleck, AZD2281). Cell proliferation was assayed by adding CCK8 reagent (Beyotime) to each well followed by incubation at 37 °C for 2 h. The absorbance was measured at 490 nm and the data were analyzed by the Student’s t test for two groups and by ANOVA for multiple groups. *p* < 0.05 was considered significant.

### HR reporter assay

U2OS cells integrated with DR-GFP cassettes were used for the analysis of HR efficiency. For analysis of HR efficiency in MDA-MB-436 cells, cells were transfected with DR-GFP plasmids and selected by puromycin. I-SceI induced expression of GFP in stable cell line was confirmed by WB. Cells transfected with the indicated siRNA or plasmids were then transfected with I-Scel plasmid. 48 h after transfection, cells were harvested and GFP positive cells were subjected to FACS analysis. HR efficiency was expressed as the mean ± SD of three independent experiments. The Student’s t test and ANOVA were used for data analysis. *p* < 0.05 was considered significant.

### Metaphase spreads assay

HeLa cells were treated with 5 mM hydroxyurea for 4 h and then incubated with 0.2 μg/ml Nocodazole for 16 h, and then harvested by centrifugation. The cell pellet was resuspended in 5 ml pre-cold 0.56%KCl solution and incubated at 37 °C for 15 min. After centrifugation the supernatant was discarded and the cells were fixed in 5 ml pre-cold fixation buffer (methanol: glacial acetic acid, v/v = 3:1). Cells were then collected and resuspended in 50–100 μl fixation buffer. 10 ~ 20 μl of the cell suspension was spread onto pre-cold slides. Let the slides dried, and stained with DAPI. At least 100 metaphase spreads were examined for each aberration. Data were expressed as mean ± SD of three independent experiments.

### Immunohistochemistry staining

Breast cancer tissue microarray (TMA) were purchased from Shanghai Outdo Biotech Company and Alena Biotech, which contains 45 pairs of breast cancer tissues together with matched adjacent normal breast tissues, 50 of invasive ductal carcinoma tissues, 307 of breast tumor tissues with different clinicopathological TNM stages and subtypes of breast cancer specimens, respectively. IHC staining was performed according to manufacturer’s instruction. For antigen retrieval, the slides were incubated in citrate buffer (Solarbio C1032) at 95 °C for 20 min. For blocking endogenous peroxidases and nonspecific binding of antibodies, 3% hydrogen peroxide and 10% goat serum were used. The slides were then probed with antibodies against BRCA1 (1:200 dilution; Abcam, ab16780), USP4 (1:300 dilution; Proteintech, 66822) at 4 °C overnight, washed and then incubated with the secondary antibody for 30 min, stained with 3,3-diaminobenzidine for 5 min. Both the intensity and percentage of positively stained tumor cells were calculated to generate an H-score, which was expressed as follows: H-score = ΣPi (*i* + 1), in which *i* stands for intensity, and Pi stands for the percentage, of the stained tumor cells (negative staining = 0; weak staining = 1, moderate staining = 2, strong staining = 3).

### Statistical analysis

All data are analyzed by Student’s *t* tests or ANOVA and presented as mean ± standard deviation. Statistical calculations were performed using GraphPad Prism 8.0. *P* < 0.05 is considered statistically significant.

### Supplementary information

Supplementary Information

Reporting-summary

## Data Availability

Data on the expression and copy number analysis of USP4 genomic alterations were obtained from cBioPortal database (https://www.cbioportal.org/). Correlation of USP4 expression and overall survival (OS) in human breast cancers were obtained from Kaplan-Meier Plotter (https://kmplot.com/analysis/). The data in this study are available in the manuscript and the supplementary files.
