# Supplementary material for: The deubiquitinating enzyme USP4 regulates BRCA1 stability and function
Source: NPJ Breast Cancer. 2024 May 11;10:35. doi: 10.1038/s41523-024-00641-7 (PMC11088691; doi:10.1038/s41523-024-00641-7)

**The deubiquitinating enzyme USP4 regulates BRCA1 stability and function**

## 1. Supplementary Figures & Legends

### Supplementary Figure 1

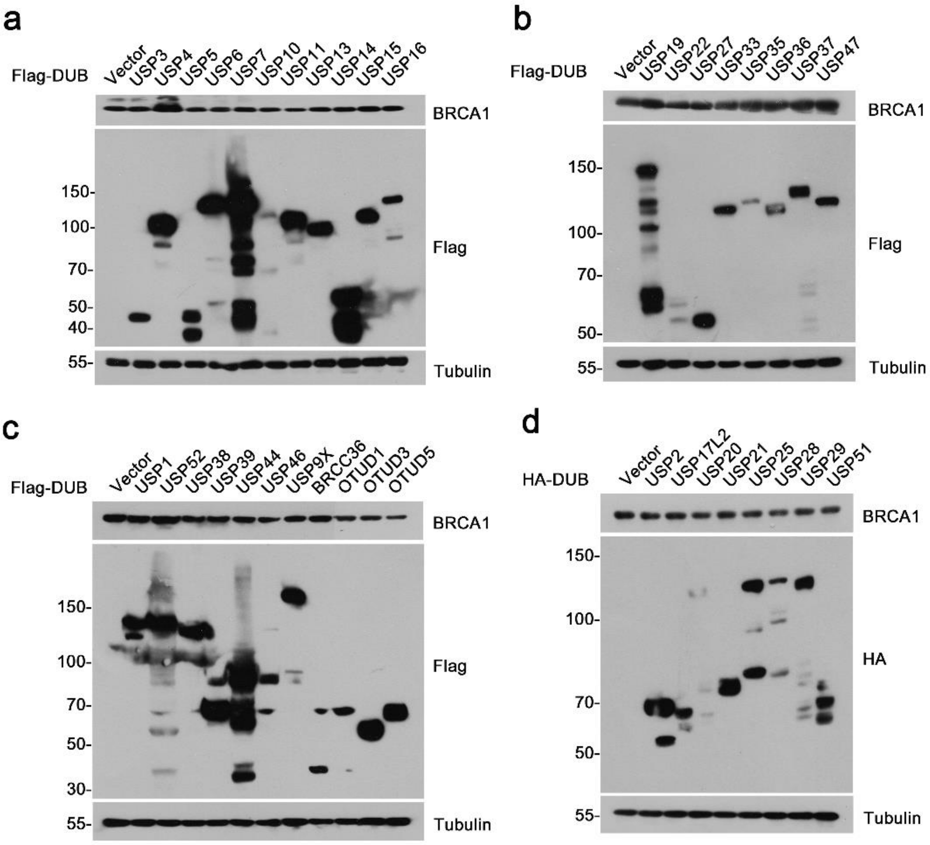


**Supplementary Figure 1. USP4 stabilizes BRCA1.**

**(a-d)** Screening for the deubiquitinating enzymes of BRCA1. The indicated DUBs were transfected into HEK293T cells. Forty-eight hours later, cell lysates were subjected to Western blotting (WB) and then normalized to the control vector.

**Supplementary Figure 2**

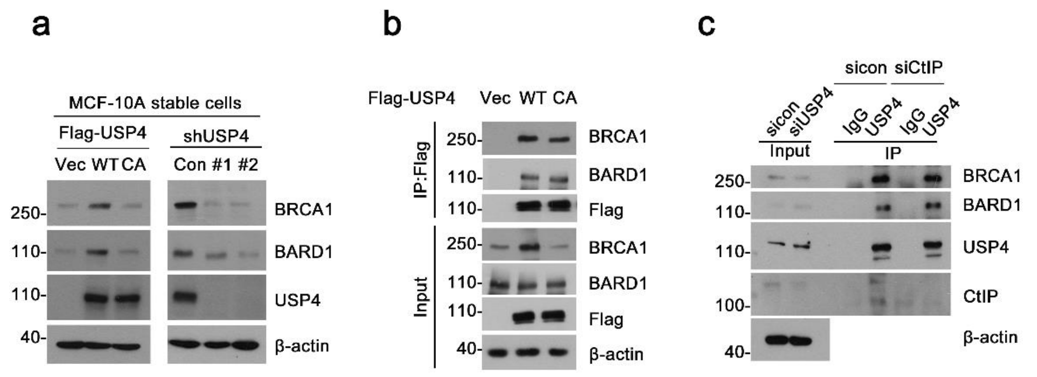


**Supplementary Figure 2. USP4 interacts with BRCA1.**

**(a)** Validation of MCF-1-A stable cell lines by WB. **(b)** Flag-USP4 interacts with endogenous BRCA1 in MCF-10A cells. Flag-USP4 WT or CA were used for Flag-IP, followed by IB. **(c)** Depletion of CtIP by siRNA in MCF-10A cells, IP with anti-USP4 antibody and IB with anti-USP4 or BRCA1 antibody, respectively, IgG IP is a negative control

**Supplementary Figure 3**

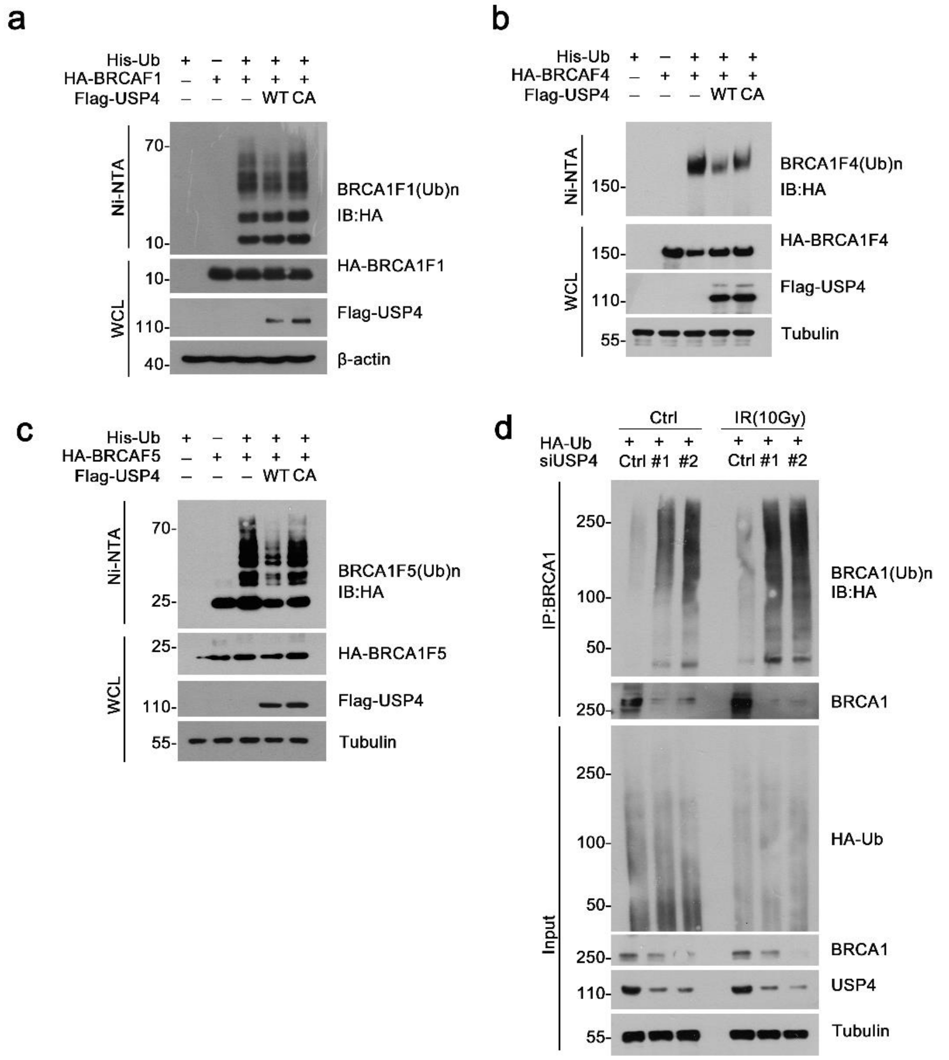


**Supplementary Figure 3. USP4 deubiquitylates BRCA1.**

**(a-c)** BRCA1 fragment constructs ubiquitylation was performed in HEK293T cells transfected with BRCA1F1 (a) or BRCA1F4 (b) or BRCA1F5 (c) together with control, USP4 WT, or CA mutant. **(d)** Ni-NTA pull-down were performed to investigate the ubiquitylation of BRCA1 after X-ray treatment.

**Supplementary Figure 4**

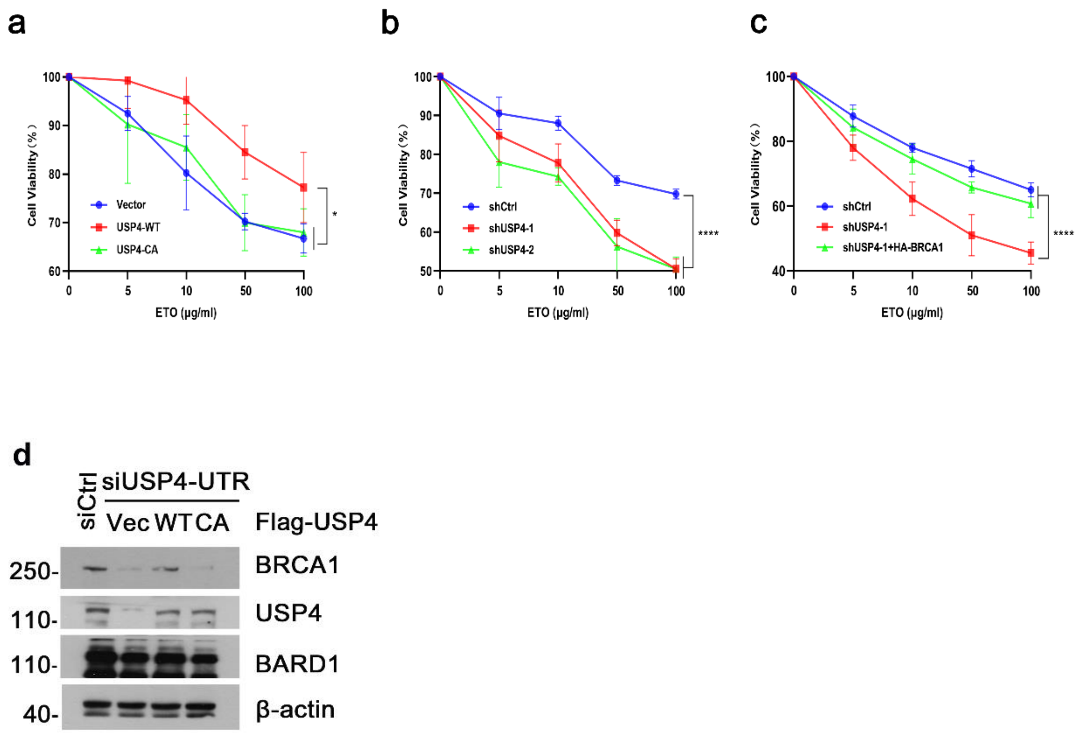


**Supplementary Figure 4. USP4 regulates the cellular functions of BRCA1 in DNA damage repair.**

**(a-b)** Cell sensitivity of MDA-MB231 cells stably expressing control, USP4 WT or CA mutant (a) or control, USP4 shRNAs (b) when treated with etoposide. **(c)** Cell sensitivity of MDA-MB231 cells stably depleting USP4 transfected with HA-BRCA1 or control plasmids when treated with etoposide. (d) Validation of DR-U2OS cells with the indicated constructs transfected by western blot.

## Supplementary Figure 5

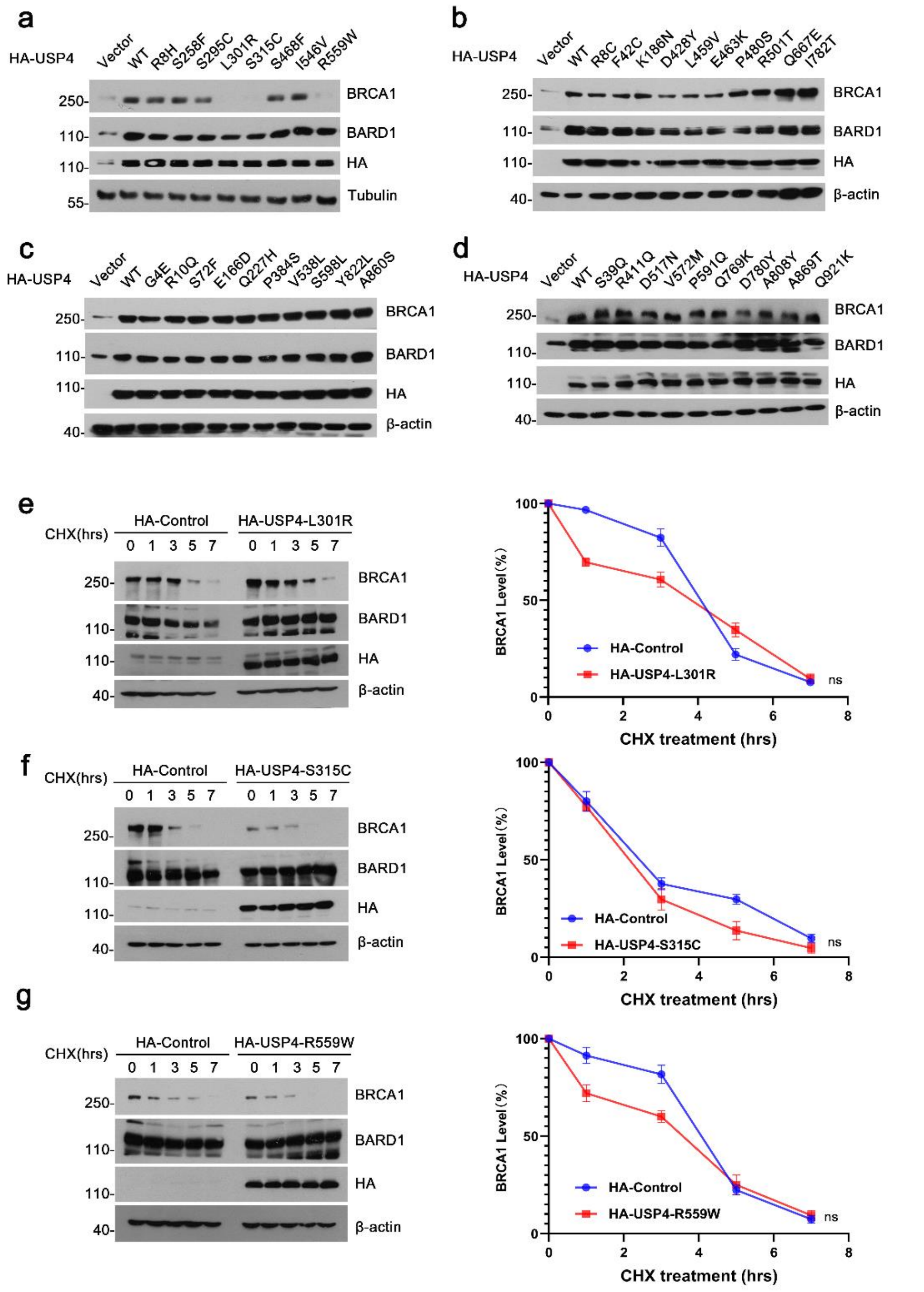


**Supplementary Figure 5. Identification of USP4 loss-of-function mutations in human cancers.**

**(a-d)** Expression of USP4 mutation caused the reduction of BRCA1. Mutations of USP4, which have been reported in the cBioPortal database were cloned into the pHA-CMV vector and transfected into HEK293T cells. Cell lysates were analyzed by WB to show BRCA1 protein levels. Wild-type USP4 was used as the positive control. **(e-g)** HEK293T cells

transfected with HA-USP4-WT or L301R, S315C, or R559W mutants were treated with 10 μg/ml CHX for the indicated time intervals, and protein levels of endogenous BRCA1 and ectopic USP4 were analyzed by WB. Quantification of BRCA1 levels relative to β-actin is shown on the right. Data are the representative results of three independent experiments. *$p<0.05$, ns non-specific, two-way ANOVA test.

## Supplementary Figure 6

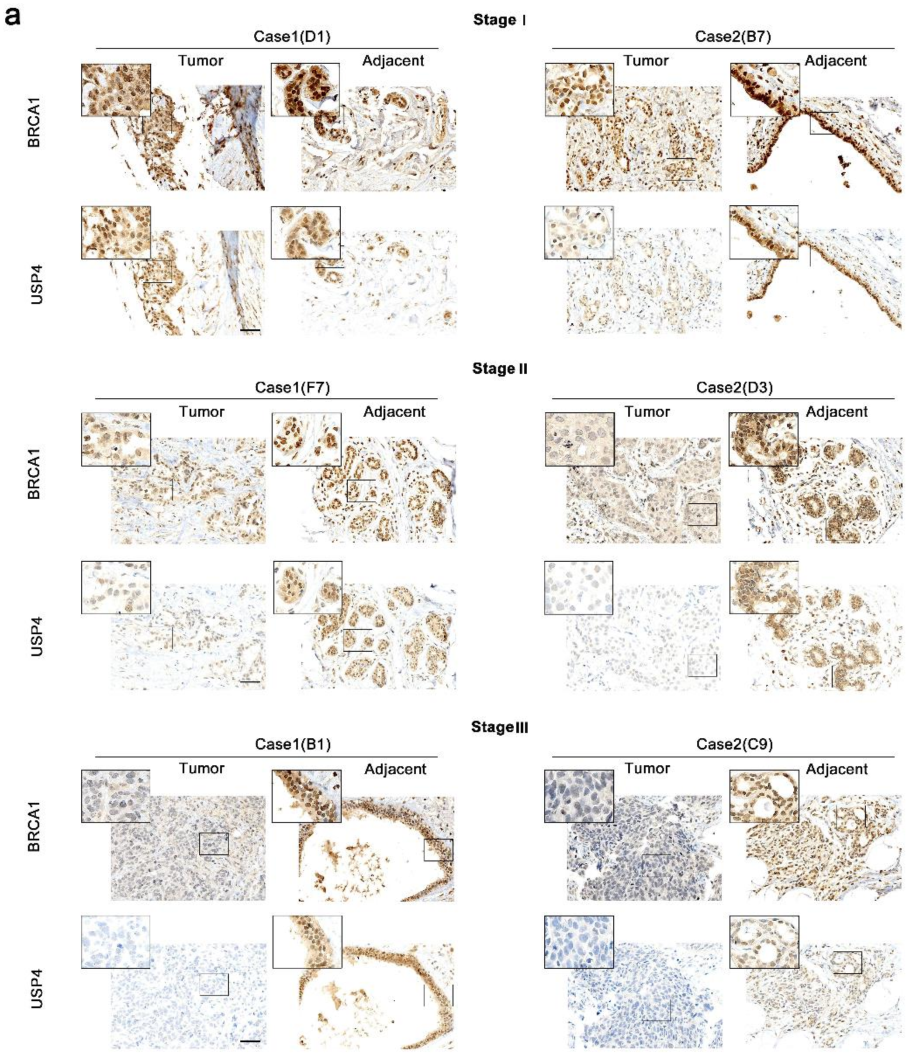

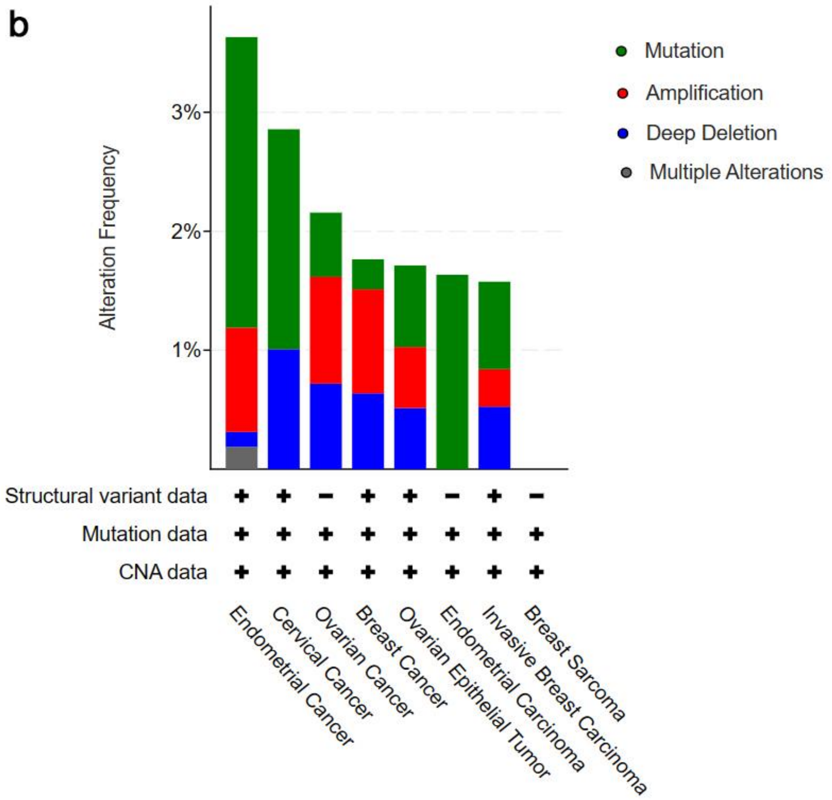


**Supplementary Figure 6. USP4 protein is downregulated in human breast cancer and positively correlates with BRCA1 protein level.**

**(a)** Association between BRCA1 and USP4 protein level was evaluated by IHC in 90 cases of breast cancer tissues within different clinicopathological TNM stages. Representative photographs of BRCA1 and USP4 immunoreactivity in normal adjacent breast tissues and paired cancer tissues with different TNM stages in a breast tissue microarray are shown. Scale bar, 200 μm. **(b)** USP4 alterations in gynecological cancers. 49 studies (18486 samples) of breast, ovarian, cervical and endometrial cancers from cBioPortal database (https://www.cbioportal.org) were selected and plotted.

2. Original WB blot Scans

Figure 1b

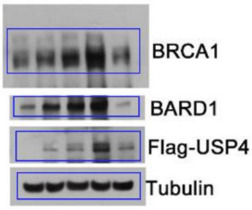


Figure 1c

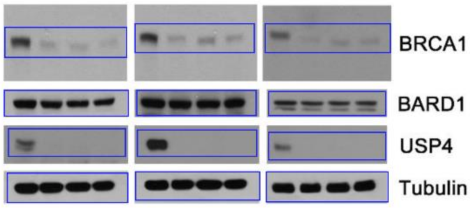


Figure 1d

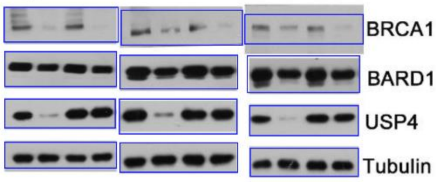


Figure 1e

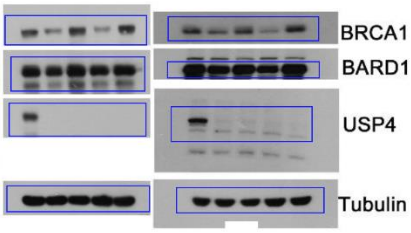


Figure 1f

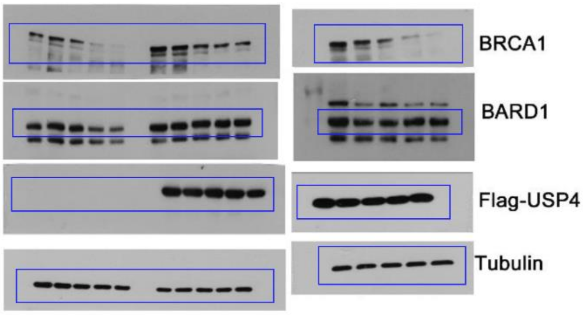


Figure 1g

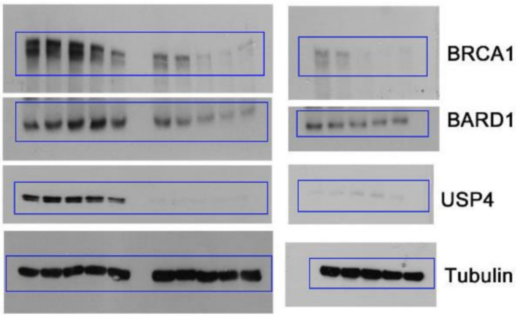

Figure 2a

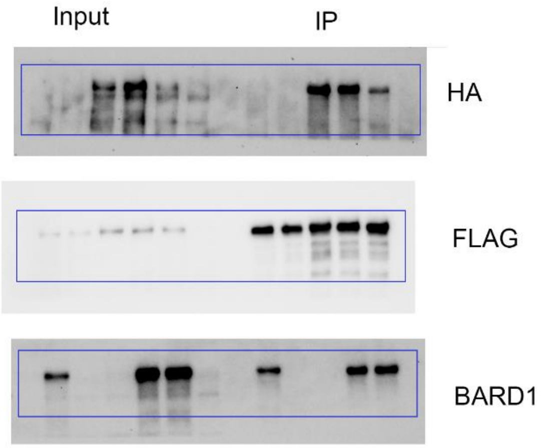


Figure 2b

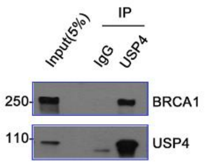


Figure 2c

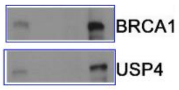


Figure 2d

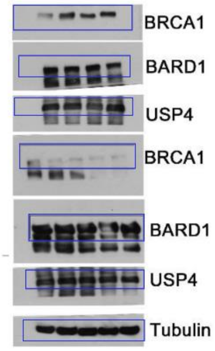


Figure 2e

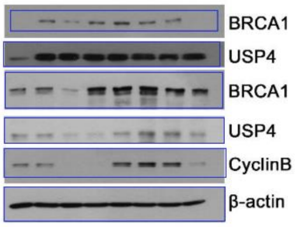


Figure 2g

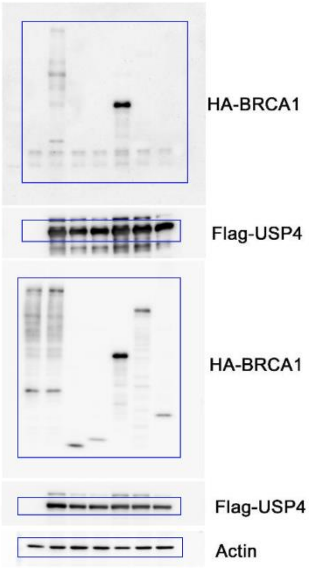


Figure 2f

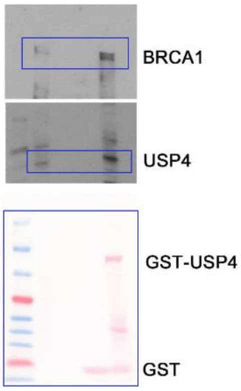


**Figure 2i**

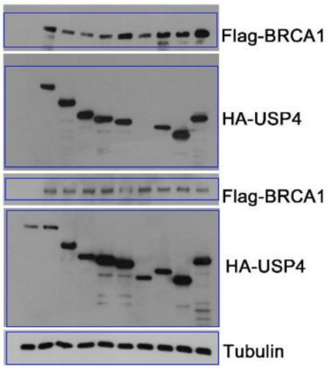

Figure 3a

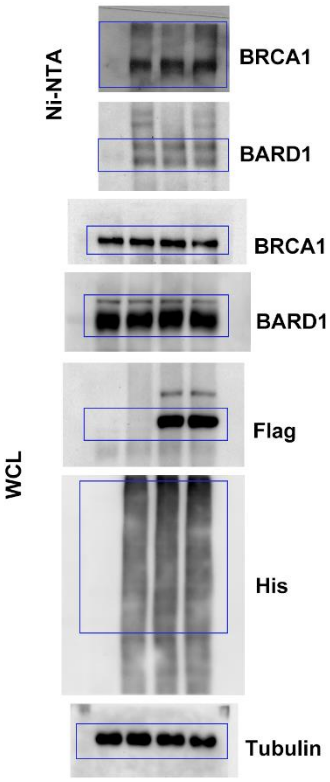


Figure 3b

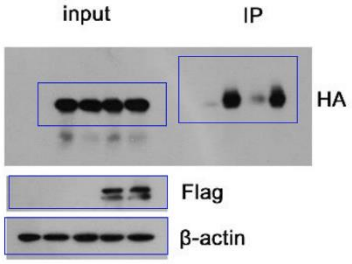


Figure 3c

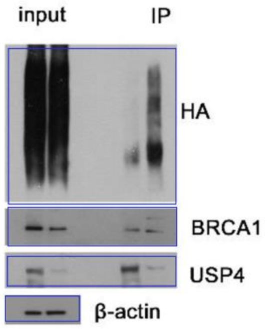


Figure 3d

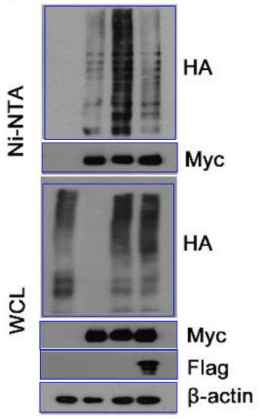


Figure 3e

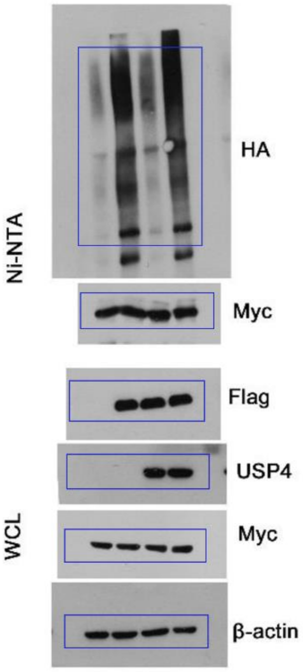


Figure 3f

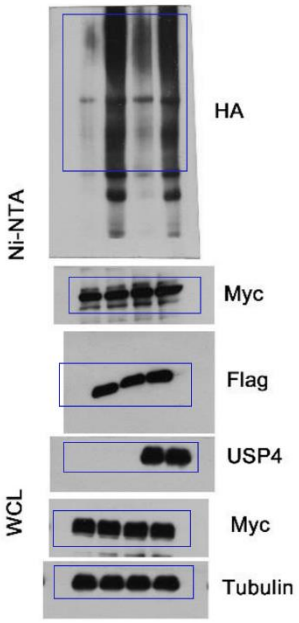


Figure 3g

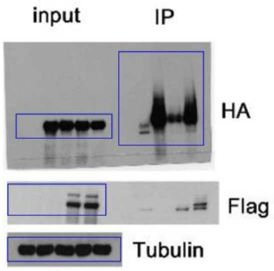


Figure 3h

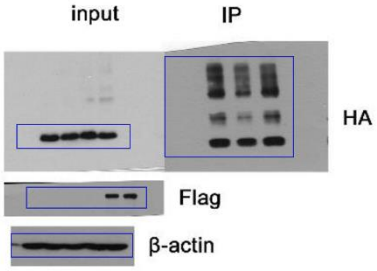


Figure 3i

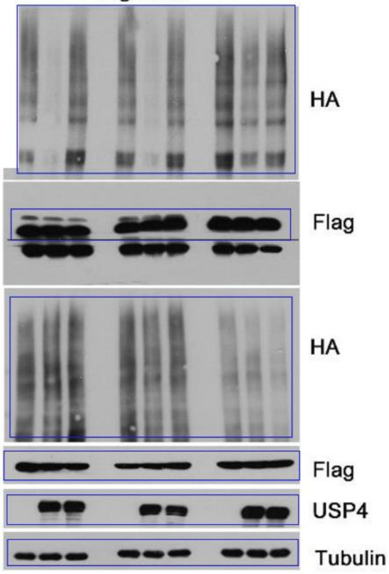

Figure 4i

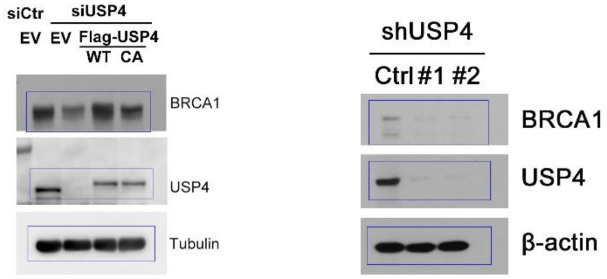


Figure 5a

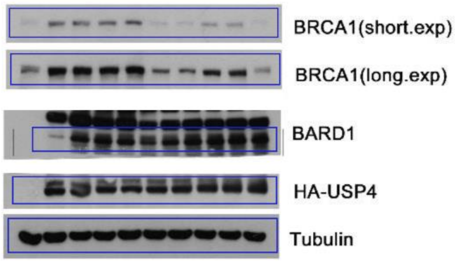


Figure 5b

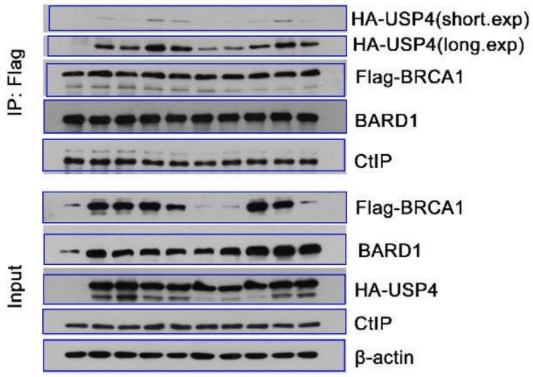


Figure 5c

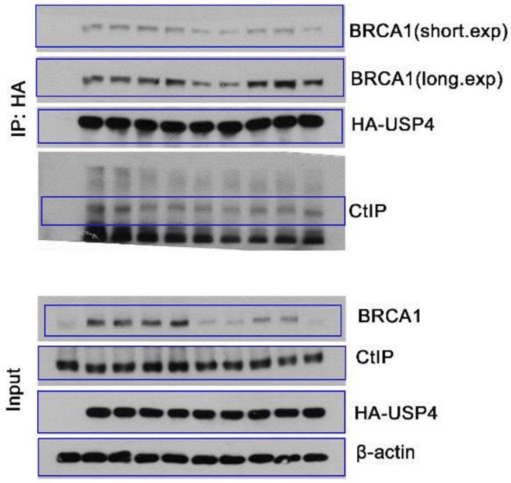


Figure 5d

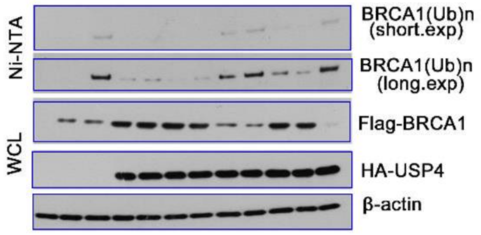


Figure 5e

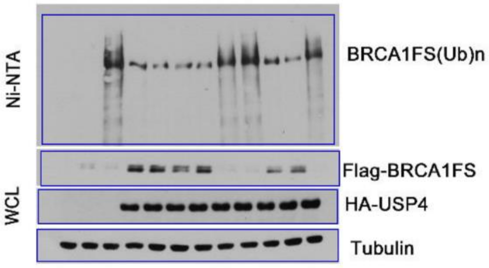


Figure 5f

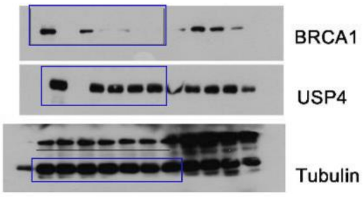

## Figure 6e

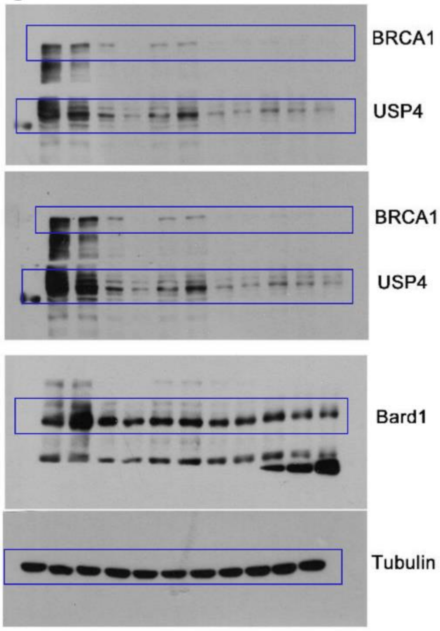

Supplement: Supplementary file 1 — Supplementary Information [file 41523_2024_641_MOESM1_ESM.pdf]
