# Supplementary material for: The deubiquitinating enzyme USP4 regulates BRCA1 stability and function
Source: NPJ Breast Cancer. 2024 May 11;10:35. doi: 10.1038/s41523-024-00641-7 (PMC11088691; doi:10.1038/s41523-024-00641-7)
Supplement: Supplementary file 2 — Reporting-summary [file 41523_2024_641_MOESM2_ESM.pdf]

nature portfolio

Corresponding author(s): Genze Shao

Last updated by author(s): Mar 15, 2024

Reporting Summary

Nature Portfolio wishes to improve the reproducibility of the work that we publish. This form provides structure for consistency and transparency in reporting. For further information on Nature Portfolio policies, see our Editorial Policies and the Editorial Policy Checklist.

Statistics

For all statistical analyses, confirm that the following items are present in the figure legend, table legend, main text, or Methods section.

n/a	Confirmed	
☐	☒	The exact sample size (*n*) for each experimental group/condition, given as a discrete number and unit of measurement
☒	☐	A statement on whether measurements were taken from distinct samples or whether the same sample was measured repeatedly
☐	☒	The statistical test(s) used AND whether they are one- or two-sided<br>*Only common tests should be described solely by name; describe more complex techniques in the Methods section.*
☐	☒	A description of all covariates tested
☒	☐	A description of any assumptions or corrections, such as tests of normality and adjustment for multiple comparisons
☐	☒	A full description of the statistical parameters including central tendency (e.g. means) or other basic estimates (e.g. regression coefficient) AND variation (e.g. standard deviation) or associated estimates of uncertainty (e.g. confidence intervals)
☐	☒	For null hypothesis testing, the test statistic (e.g. *F*, *t*, *r*) with confidence intervals, effect sizes, degrees of freedom and *P* value noted<br>*Give P values as exact values whenever suitable.*
☒	☐	For Bayesian analysis, information on the choice of priors and Markov chain Monte Carlo settings
☒	☐	For hierarchical and complex designs, identification of the appropriate level for tests and full reporting of outcomes
☒	☐	Estimates of effect sizes (e.g. Cohen's *d*, Pearson's *r*), indicating how they were calculated

Our web collection on statistics for biologists contains articles on many of the points above.

Software and code

Policy information about availability of computer code

Data collection	NA
Data analysis	NA

For manuscripts utilizing custom algorithms or software that are central to the research but not yet described in published literature, software must be made available to editors and reviewers. We strongly encourage code deposition in a community repository (e.g. GitHub). See the Nature Portfolio guidelines for submitting code & software for further information.

Data

Policy information about availability of data

All manuscripts must include a data availability statement. This statement should provide the following information, where applicable:

- Accession codes, unique identifiers, or web links for publicly available datasets
- A description of any restrictions on data availability
- For clinical datasets or third party data, please ensure that the statement adheres to our policy

Data on expression and copy number analysis of USP4 genomic alterations in cBioPortal database (https://www.cbioportal.org/). Correlation of USP4 expression and overall survival (OS) in human breast cancers from Kaplan-Meier Plotter (https://kmplot.com/analysis/). The data in this study are available in the manuscript and the supplementary files

Research involving human participants, their data, or biological material

Policy information about studies with human participants or human data. See also policy information about sex, gender (identity/presentation), and sexual orientation and race, ethnicity and racism.

Reporting on sex and gender	NA
Reporting on race, ethnicity, or other socially relevant groupings	NA
Population characteristics	NA
Recruitment	NA
Ethics oversight	NA

Note that full information on the approval of the study protocol must also be provided in the manuscript.

Field-specific reporting

Please select the one below that is the best fit for your research. If you are not sure, read the appropriate sections before making your selection.

☒ Life sciences ☐ Behavioural & social sciences ☐ Ecological, evolutionary & environmental sciences

For a reference copy of the document with all sections, see nature.com/documents/nr-reporting-summary-flat.pdf

Life sciences study design

All studies must disclose on these points even when the disclosure is negative.

Sample size	NA
Data exclusions	NA
Replication	NA
Randomization	NA
Blinding	NA

Reporting for specific materials, systems and methods

We require information from authors about some types of materials, experimental systems and methods used in many studies. Here, indicate whether each material, system or method listed is relevant to your study. If you are not sure if a list item applies to your research, read the appropriate section before selecting a response.

Materials & experimental systems

n/a	Involved in the study	
☐	☒	Antibodies
☐	☒	Eukaryotic cell lines
☒	☐	Palaeontology and archaeology
☒	☐	Animals and other organisms
☒	☐	Clinical data
☒	☐	Dual use research of concern
☒	☐	Plants

Methods

n/a	Involved in the study	
☒	☐	ChIP-seq
☐	☒	Flow cytometry
☒	☐	MRI-based neuroimaging

Antibodies

Antibodies used	All antibodies used were commercially available and described in the methods.
Validation	Information including data sheet for each of the antibodies was provided by the manufacturers. All the antibodies were validated by the manufacturers.

Eukaryotic cell lines

Policy information about cell lines and Sex and Gender in Research

Cell line source(s)	HEK293T, HeLa, MCF-7, MDA-MB231, MCF-10A and MCF-10F were purchased from ATCC
Authentication	All cell lines used in this study have been verified by STR. The STR confirms that all the cell lines used are authentic.
Mycoplasma contamination	No mycoplasma contamination
Commonly misidentified lines (See ICLAC register)	NA

Plants

Seed stocks	NA
Novel plant genotypes	NA
Authentication	NA

Flow Cytometry

Plots

Confirm that:

☒ The axis labels state the marker and fluorochrome used (e.g. CD4-FITC).

☒ The axis scales are clearly visible. Include numbers along axes only for bottom left plot of group (a 'group' is an analysis of identical markers).

☒ All plots are contour plots with outliers or pseudocolor plots.

☒ A numerical value for number of cells or percentage (with statistics) is provided.

Methodology

Sample preparation	The collected cells were washed with 1× PBS. Centrifuge the cells at 1300–1500 revolutions per minute (rpm) for 5 mins. Discard the supernatant and add 0.5 mL 1× PBS to each tube and resuspend the cells gently by pipetting up and down. To fix the cells add 4.5 mL ice-cold 100% methanol to the cell suspension and incubate the cells at −20 °C refrigerator for 20 mins. Centrifuge the cells at 1300–1500 rpm for 5 mins. Centrifuge the cells at 1300–1500 rpm for 5 mins. Discard the supernatant and add 1 mL Ribonuclease (RNase) buffer to each tube and resuspend the cells gently by pipetting up and down. Incubate the cells on ice for 30 mins. Centrifuge the cells at 1300–1500 rpm for 5 mins. Discard the supernatant and add 1 mL PI solution to each tube and resuspend the cells gently by pipetting up and down. Incubate the cells on ice for 5–10 mins.
Instrument	caliber2
Software	BD FACSDiva
Cell population abundance	10000
Gating strategy	Cells are first gated on the basis of Forward (FSC) and side scatter (SSC), which give an idea of the size and granularity of the cells respectively.

☐ Tick this box to confirm that a figure exemplifying the gating strategy is provided in the Supplementary Information.
